# Covalent organic framework-based electrochemical nanosensing: an emerging paradigm for early cancer diagnosis and longitudinal surveillance

**DOI:** 10.1186/s12951-025-03988-6

**Published:** 2026-01-01

**Authors:** Yue Zhang, Shuyi Chen, Jie Ma, Xiaobin Zhou, Xinchen Sun, Chenglin Zhou

**Affiliations:** 1 Clinical Medical Laboratory Center, Gaogang Branch, Taizhou School of Clinical Medicine, Nanjing Medical University, The Affiliated Taizhou People’s Hospital of Nanjing Medical University, Taizhou, 225300 China; 2 Clinical laboratory department, The Second Affiliated Hospital of Anhui Medical University, Hefei, 230601 China

**Keywords:** Covalent organic frameworks, Electrochemical nanosensing, Cancer diagnosis and surveillance, Liquid biopsy, Clinical application

## Abstract

The management of cancer relies crucially on early diagnosis and personalized treatment. Real-time analysis of tumor markers within the tumor microenvironment via liquid biopsy opens potential pathways for effective cancer treatment and improved survival rates. Detecting low-abundance tumor markers in bodily fluids, particularly during early-stage cancer, poses significant challenges for traditional methods. Electrochemical sensors have emerged as the preferred technology for liquid biopsy. The exceptional multifunctionality of covalent organic frameworks (COFs)—novel crystalline porous organic polymer materials—has led to significant attention in electrochemical sensing; these features include tunable topologies, controllable pore sizes, and strong π-π stacking interactions. Recent advances in COF-based electrochemical sensors for liquid biopsy are summarized here, with details on COF design principles, synthesis and functionalization methods, and electrochemical reaction mechanisms. The focus is on the use of COFs as novel functional materials in electrochemical sensors for detecting tumor markers. Enhancement strategies for COF-based electrochemical sensors are also explored. An in-depth discussion on translating COF-based electrochemical sensors from laboratory achievements into clinical applications is also presented, covering the associated opportunities, challenges, and future research directions. The aim of this review is to offer concise yet profound guidance on the clinical translation of COF-based electrochemical analytical methods, which can contribute to advancing human health and precision diagnostics.

**Graphical abstract:**

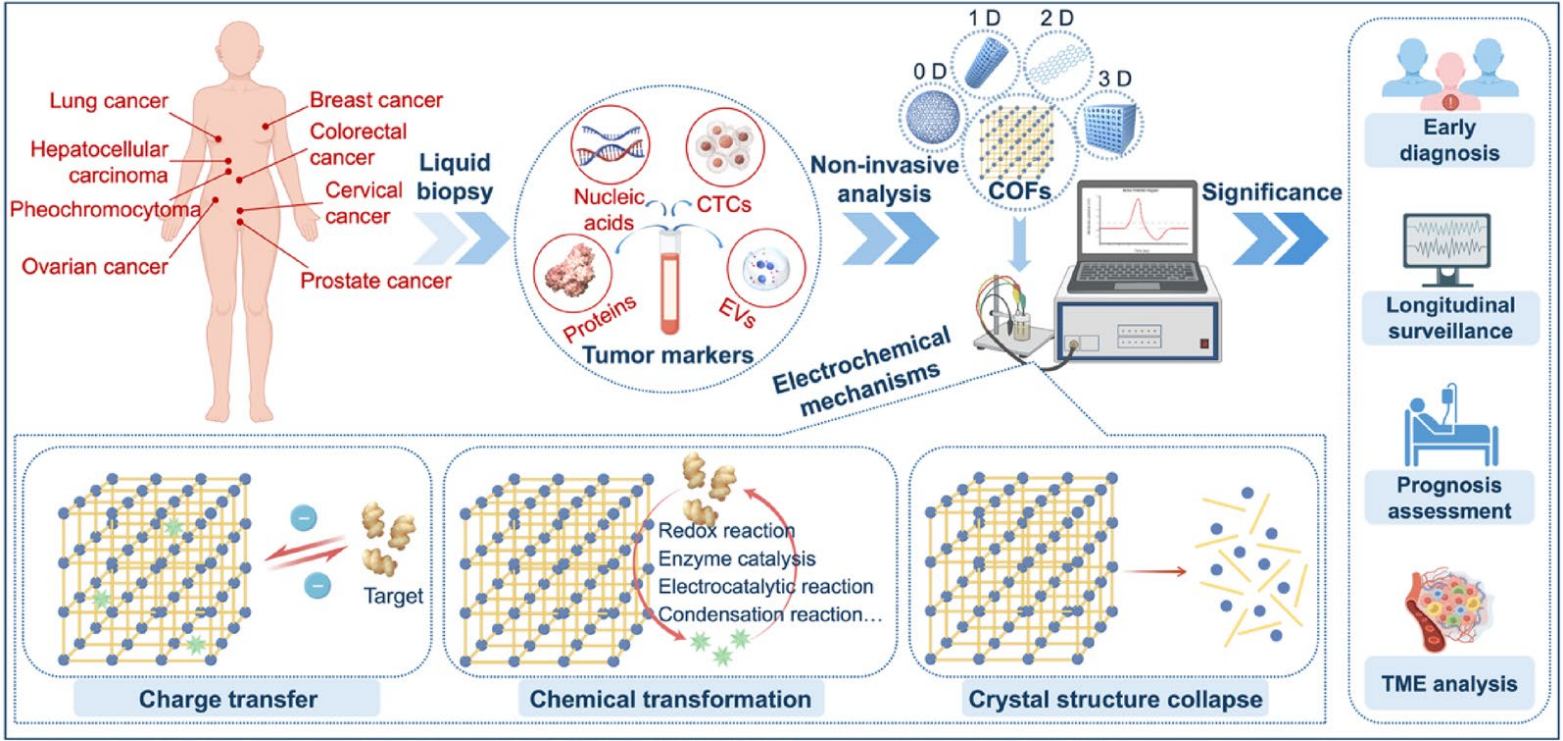

## Introduction

**Fig. 1:**
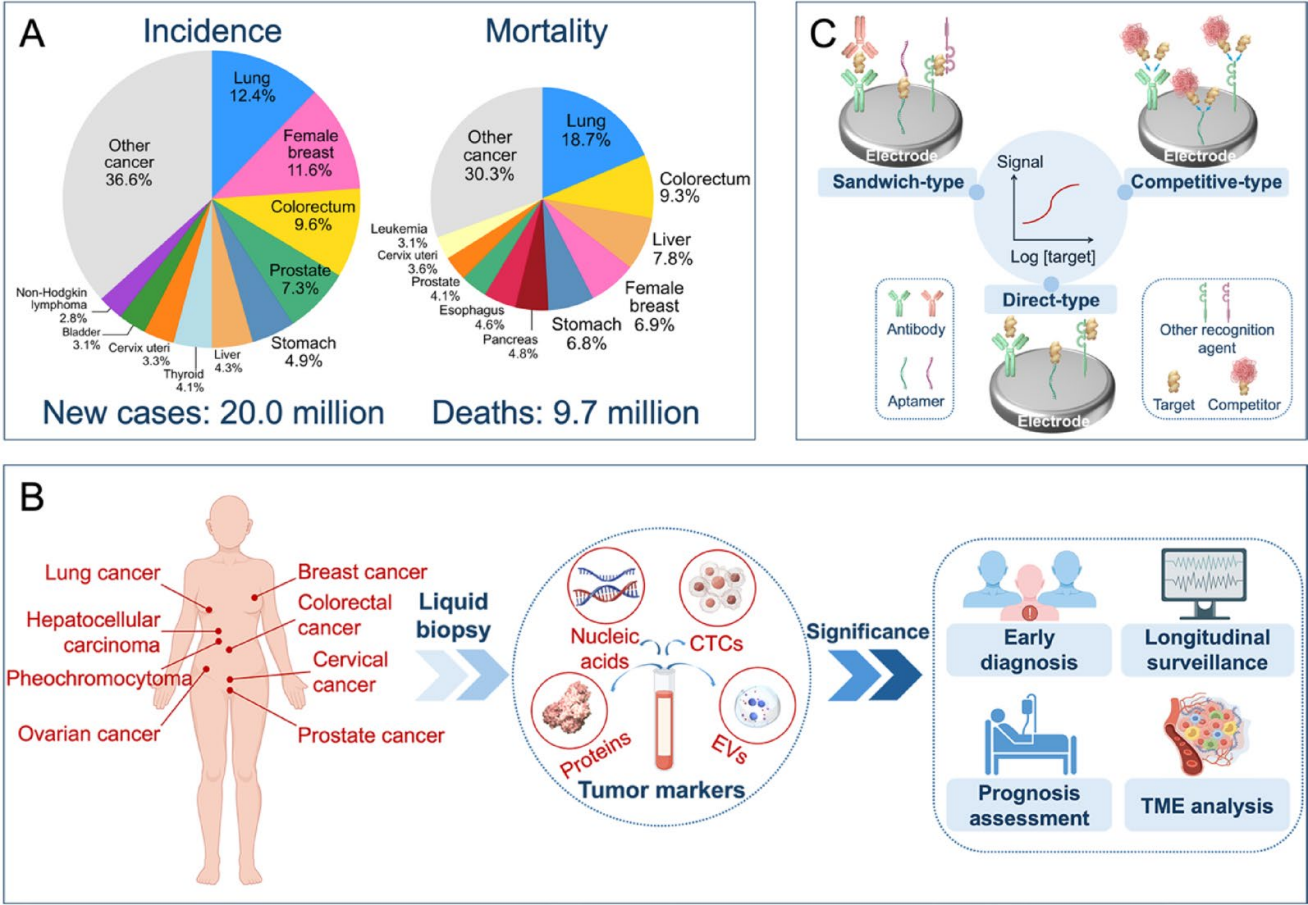
Overview highlighting the significance of early cancer diagnosis and treatment effectiveness assessment. (**A**) The number of new cancer cases and cancer-related deaths worldwide in 2022. The data are from IARC [1]. (**B**) Liquid biopsy represents a precise and reliable approach for cancer diagnosis and monitoring. (**C**) Schematic illustration of the electrochemical sensors for detecting tumor markers. The common electrochemical sensing formats are divided into direct, competitive, and sandwich assays. Created with Figdraw

Malignant tumors pose a major issue for both global public health and economics. The International Agency for Research on Cancer (IARC) released recent statistics indicating 20 million new cancer cases worldwide in 2022. These cases resulted in 9.7 million cancer-related deaths [1]. This mortality rate constitutes approximately one-quarter of all deaths from non-communicable diseases globally and presents a significant challenge to worldwide health (Fig. 1A) [2]. A significant difference exists in the five-year survival rates between early-stage and late-stage cancer patients, a difference that is particularly evident in lung, breast, and cervical cancers. A primary challenge is that most cancers lack symptomatic lesions during their early stages. Therefore, the absence of effective screening methods leads to the diagnosis of half of all cancer cases at an advanced stage [3, 4]. Improving cancer patient survival rates therefore depends on early diagnosis and effective intervention. For this reason, developing simple and efficient strategies for cancer diagnosis and treatment effectiveness assessment holds important clinical significance.

As a fundamental characteristic of cancer, tumor heterogeneity involves differences in genetic and phenotypic features both in individual tumors and among different tumors. The presence of complex tumor heterogeneity directly influences treatment outcomes and patient prognosis [5–7]. Traditional cancer diagnosis and monitoring currently rely on methods such as pathological biopsy and various imaging examinations (e.g., computed tomography [CT], positron emission tomography-CT [PET-CT], and ultrasound) [8–11]. In pathological diagnosis, an operator must observe suspicious cancer lesions under a microscope; this process can be both time-consuming and susceptible to operator bias. Pathological biopsy, despite being the “gold standard” for diagnosis, has limitations, as it can produce false-negative results because of tumor microenvironment (TME) heterogeneity and is often unsuitable for repeated invasive biopsies due to associated risks and patient discomfort [12]. Imaging examinations also present several drawbacks, such as radioactivity, long intervals between tests, low specificity, and high false-positive rates [13]. This highlights the clear and evident need for convenient, efficient, accurate, non-invasive, and patient-friendly methods to enable early cancer diagnosis and dynamic surveillance.

Liquid biopsy represents a powerful alternative that involves analyzing specific components in body fluids. This approach holds several key advantages, including being non-invasive, non-radiative, simple, efficient, and patient-friendly [14]. Multiple comparisons and analyses of various tumor markers in peripheral blood can circumvent the issue of TME heterogeneity. This allows for the provision of real-time disease information and facilitates longitudinal surveillance, which supports the early diagnosis and personalized treatment of cancer [15, 16]. These targets of detection, known as tumor markers, are substances that tumor cells synthesize themselves or that the body produces abnormally in response [17, 18]. They consist of traditional markers such as carcinoembryonic antigen (CEA), carbohydrate antigen 15 − 3 (CA15-3), prostate-specific antigen (PSA), and carbohydrate antigen 125 (CA125) [19–21]. Besides, they include novel markers such as circulating tumor deoxyribonucleic acid (ctDNA), circulating tumor cells (CTCs), extracellular vesicles (EVs), sperm protein 17 (Sp17), and programmed cell death-ligand 1 (PD-L1) [22–25]. Analysis through testing not only facilitates early diagnosis but also allows biomarker results to be integrated for monitoring treatment effectiveness, assessing prognosis, and analyzing the tumor immune microenvironment (Fig. 1B). However, commonly utilized liquid biopsy methods, including radioimmunoassay and enzyme-linked immunosorbent assay (ELISA), are often limited by time, space, and cost constraints. These methods hold drawbacks such as cumbersome procedures, bulky equipment, and high analytical costs, which render them unsuitable for use in remote areas. These limitations significantly restrict the use of tumor markers for early cancer diagnosis and dynamic monitoring [26–28].

As the patterns and strategies of liquid biopsy have been constantly evolving, electrochemical detection methods are developed accordingly to overcome these challenges. These methods achieve accurate measurement of targets by utilizing antibodies, aptamers, and other agents to specifically recognize targets, converting biological reaction signals into electrical signals [29, 30]. Compared to traditional analytical methods, electrochemical sensors offer the advantages of miniaturization, low cost, simple operation, and high sensitivity, leading to their wide application in disease diagnosis and healthcare [31, 32].The common electrochemical sensing formats are shown in Fig. 1C. For instance, the use of electrochemical sensors for dopamine detection can be adopted in assessing hypertension, heart failure, and Alzheimer’s disease [33]. Notably, electrochemical sensors can be integrated with microfluidics or wearable platforms to advance point-of-care testing (POCT) [34–36]. Electrochemical sensors based on screen-printed carbon electrodes (SPCEs) can detect menthol and tumor protein 53 (TP53) with ultra-high sensitivity to effectively predict epileptic seizures and diagnose esophageal and oral cancers [37–39]. Moreover, personalized health management may be realized with a wearable sweat sensor for detecting lactate thresholds with an electrochemical colorimetric platform [40]. Therefore, as a powerful alternative to traditional liquid biopsy methods, it is necessary for electrochemical sensors to fulfil higher performance requirements.

The recently rapid development of nanomaterials have afforded novel opportunities for electrochemical sensors performance enhancement [41, 42]. Covalent organic frameworks (COFs) are one such development. These new crystalline porous organic polymers are extensively applied in catalysis, separation, and adsorption due to their desirable properties, such as high porosity, a highly ordered structure, controllable pore size, and easy functionalization [43–46]. In the field of electrochemical sensing, they have likewise demonstrated significant application potential. COFs in their native form can act as signal amplifiers or carriers for anchoring metal nanoparticles for modification. They may also be integrated as reaction carriers or enrichment units, which makes them ideal enhancers for sensors [47–50]. Researchers have customized different functionalized COFs for the electrochemical detection of DNA methyltransferase (MTase) activity and salivary microRNAs (miRNAs), supporting the creation of treatment strategies for methylation-related diseases and diagnostic strategies for mild traumatic brain injury [51, 52]. A study by Cui et al. utilized photoactive COFs to regulate a zinc-air battery self-powered electrochemical sensor for the ultra-sensitive detection of the H1N1 influenza virus haemagglutinin protein [53]. In another example, a fluorescence/electrochemical dual-mode sensor was developed by combining COFs with multi-space-located DNA walkers, which employed a walk-retrieve-convert strategy for the accurate measurement of nucleocapsid proteins [54]. The “structure-function integration” characteristics of COFs render it excellent candidate materials for modifying electrochemical sensors. Its highly conductive conjugated skeleton, redox activity of functional groups, and unique anti-interference capabilities provide an efficient solution for the analysis of trace tumor markers in complex media, indicating broad application prospects in electrochemical sensing.

The development of COF-based electrochemical sensors for early cancer diagnosis and treatment efficacy assessment represents a burgeoning area of research. This review offers a systematic summary of progress achieved in the last five years on COF-based electrochemical sensors for cancer liquid biopsy. It covers strategies for constructing COFs and their electrochemical reaction mechanisms. Particular emphasis is accorded to the roles of COFs when integrated with methods such as enzyme or enzyme-like catalytic amplification, electrocatalytic reactions, DNA strategies, magnetic separation, and nano-functionalization technologies for electrochemical analysis. A unique aspect of this review is its exploration of the clinical translation prospects for COF-based electrochemical sensors in cancer diagnosis and surveillance. The discussion covers the advantages and challenges associated with COFs, the translational pathway from laboratory to clinic, regulatory approval processes, and practical barriers to deployment. In addition, the text explores interdisciplinary collaborations, such as integrating these sensors with microfluidic or wearable platforms and fusing them with machine learning (ML)/artificial intelligence (AI), and also proposes future directions for research. The aim of this review is to advance COF-based electrochemical analytical methods by offering researchers and clinicians novel research directions and clear implementation pathways. The goal is to offer more accurate and reliable approaches for diagnosing and monitoring cancer, thus significantly contributing to protecting human health and well-being.

## Design, synthesis, and electrochemical reaction mechanisms of COFs

**Fig. 2:**
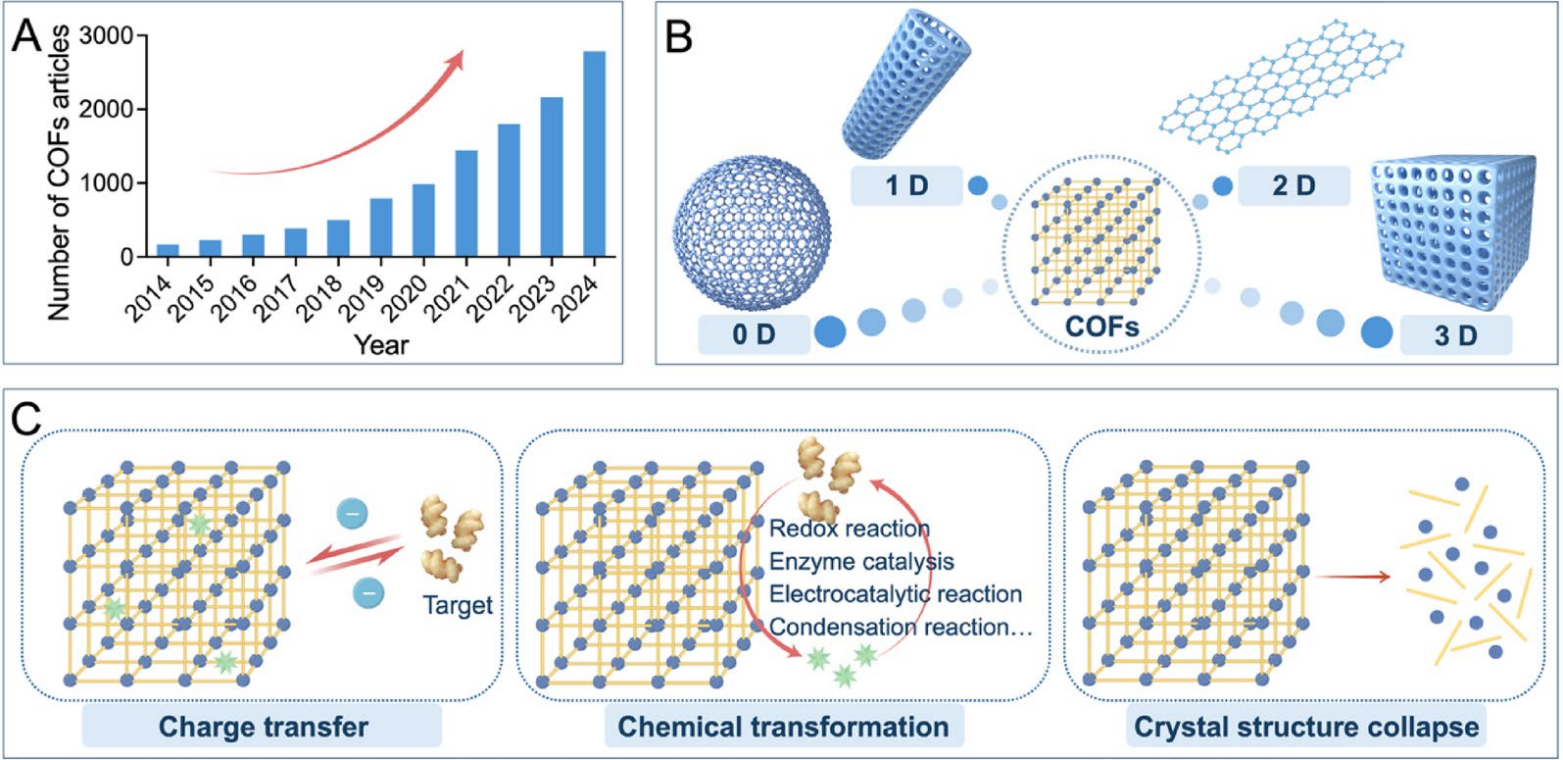
Overview of COFs. (**A**) The number of COFs-related articles per year. The data were based on the search results from Web of Science. (**B**) Typical structural forms of 0D, 1D, 2D, and 3D COFs. (**C**) The schematic reaction mechanisms of COF-based electrochemical sensors: charge transfer, chemical transformation, and crystal structure collapse. Created with Figdraw

 In 2005, the Yaghi research group first synthesized COFs utilizing dynamic covalent chemistry and topological principles [55]. These materials represent a class of crystalline porous organic structures, which are composed of light elements (C, N, O, B, Si) connected by covalent bonds (such as C-N, C-C, B-N, B-O-Si). The organic building units offer rigidity and designability, endowing COFs with exceptional orderliness, controllability, and stability [56, 57]. Accordingly of these excellent properties, the number of research results on COFs has grown steadily each year (Fig. 2A).

### Design and structure of COFs

The design of COFs finds its basis on the principle of cross-linking chemistry, where monomers with symmetric active groups polymerize according to a predefined geometric structure [58]. Structural design offers a high degree of controllability, allowing the formation of crystal frameworks with atomic-level accuracy. The geometric symmetry of the building units classifies COFs into zero-dimensional (0D), one-dimensional (1D), two-dimensional (2D), and three-dimensional (3D) forms (Fig. 2B). 0D COFs are composed of nanoparticles or nanospheres, whereas 1D COFs usually form nanofibers, nanowires, or nanorods with a sql topological structure. 2D COFs are covalently composed of π-conjugated building units. These materials feature periodic π-π stacking columns and extended π-conjugated systems that offer effective charge transport channels, endowing them with excellent electrochemical properties. In comparison, 3D COFs have greater complexity and incorporate a wide variety of building blocks, such as *sp*^*3*^ carbon, boron atoms, or silanes, which offers great possibilities for structural design [59–61]. Effective design strategies, including molecular planarity regulation, polymerization reaction optimization, and side-chain engineering, can improve COFs crystallinity. Finally, heteroatoms in the COFs structure offer a unique reaction microenvironment, enabling their use as redox-active centers or target-binding sites in electrochemical sensing.

The type of dynamic covalent bond determines the structural diversity of COFs, with the most common types being boron-, imine-, and triazine-based. Boron-based COFs, which involve the formation of borate esters, are classified into two categories. The first category is prepared through the dehydration self-condensation reaction of boric acid and produces layered structures in a staggered arrangement. This particular type of COFs has both structural order and significant thermal stability. However, the borate formation reaction is reversible, limiting water resistance, as contact with acids, alkalis, or moisture can cause hydrolysis and destroy the framework. A second type is a borate polymer with a layer-parallel stacking structure, formed through the dehydration condensation reaction of boric acid and phenol, that also exhibits thermal stability [62, 63]. Since boron-based COFs can structurally collapse from hydrolysis in acidic or alkaline conditions, researchers developed more stable imine-based COFs. These materials are formed through a Schiff base condensation reaction that creates imine (-C = N-) or hydrazine bonds, and they exhibit a persistent pore structure, a large specific surface area, adjustable pore sizes, and excellent thermal and chemical stability [64]. Stable interactions between the COFs and metal ions are promoted by the nitrogen atom in the imine bond. For this reason, imine-based COFs are commonly utilized in electrochemical sensing applications. Besides, Thomas et al. first synthesized triazine-based COFs in 2008 [65]. While these COFs have poor crystallinity and pore size distribution, their triazine framework offers excellent thermal and chemical stability. The high heteroatom content of triazine-based COFs can offer a large number of active sites, regulate the electronic structure of the benzene ring, and promote catalytic reactions [66].

### Synthesis methods and functionalization strategies of COFs

#### Synthesis of COFs

Covalent bonds connect porous polymers to form COFs, which exhibit predefined geometric structures. The accurate control of dynamic chemistry enables the synthesis of rigid COFs structures that have regular properties along with customizable chemical and physical characteristics [67]. Typical approaches to synthesis include solvothermal, ionothermal, mechanochemical, microwave-assisted, solvent-free, and interfacial methods. These methods utilize reversible reactions (such as Schiff base condensation, borate ester formation, and triazine linkage) to produce robust, crystalline porous structures, thus obtaining COFs with varied properties (such as crystallinity, porosity, and stability).

Solvothermal synthesis is the most common method because its products exhibit high crystallinity, tunable structures, and pore sizes, which renders the method suitable for large-scale production. Its primary disadvantages are a relatively long reaction time (2–9 days) and a complex process requiring high temperatures (80–200 °C) [60, 68]. In comparison, ionothermal synthesis offers a unique environment for the formation of ionic states in ionic liquids or eutectic mixtures, enabling the production of high-purity and high-yield triazine-based COFs. This method, however, requires high reaction temperatures (85–400 °C) and long reaction times (1–9 days), and has high requirements for thermal stability and poor monomer stability [69]. Mechanochemical synthesis is represented by simple, efficient (5–300 min), and environmentally friendly operation at room temperature; however, its application scope is limited, and the resulting COFs have poor structural order [70]. For various types of COFs, microwave-assisted synthesis offers a short synthesis time (30–360 min), high yield, and low cost, though a drawback is the low crystallinity of the product [71]. Solvent-free synthesis is advantageous for its environmental friendliness and high crystallinity; nonetheless, the process takes 3–5 days and requires solid catalysts under high temperatures (120–200 °C) and high-pressure conditions [72]. Finally, interfacial synthesis can restrict the growth direction of COFs crystals at the boundary of two heterogeneous interface regions, thereby offering control over their morphology and structure [73].

The diverse characteristics of these synthesis methods allow experimental personnel to select the most suitable scheme according to their specific requirements. This selection facilitates the preparation of COFs that feature an ordered and adjustable pore structure, a large specific surface area, and a predictable, structurally stable framework.

#### Functionalization of COFs

Single COFs present certain limitations in electrochemical analysis. These limitations are due to the complex matrix of electrochemical analysis and the diverse range of targets, even with the advantages of COFs over traditional nanomaterials. Functionalization strategies are therefore crucial for enhancing COFs performance and promoting their application. The introduction of nanomaterials, structural units, or active sites can achieve the hybridization or modification of COFs. This process yields functionalized COFs that exhibit significantly enhanced performance [74]. The primary functionalization strategies for COFs are surface modification, in situ growth, and self-assembly.

Surface modification is a process where residues in the COFs backbone bind to target molecules, which forms stable nanocomposites under mild conditions. The operational simplicity of surface modification typically avoids complex condition optimization [75]. This process, however, can introduce a higher risk of COFs skeleton decomposition. Therefore, this strategy is best suited for modifying COFs with stable structures, and structural integrity must be monitored during synthesis. A stable COFs scaffold can elevate the local concentration of the materials on its surface, giving the functionalized COFs superior electrochemical sensing activity compared to the original material. In addition, functionalized COFs are capable of neutralizing charges or forming hydrophobic/hydrophilic interfaces; these abilities help the COFs sustain a relatively stable structure in solution and lead to improved dispersibility. Surface modification’s advantage is the ability to achieve a decoupled design between functionality and the skeleton. This process enables the “on-demand installation” of various functional groups through accurate post-synthesis modification, which preserves the ideal crystal structure of COFs. This method offers exceptional flexibility and universality in functionalization.

In situ growth describes the formation of a new-phase material, with a specific composition or spatially ordered structure, from the material and its adjacent elements under specific conditions [76]. This method minimizes the impurities and by-products generated during synthesis. It also improves product purity and allows for the adjustment of product morphology and size by regulating growth conditions. In comparison to surface modification, this strategy exhibits greater complexity. The core breakthrough for the in situ growth method is achieving the stable confinement and ordered integration of functional units. This approach directly embeds functional species as building blocks into the skeleton during COF synthesis, which resolves issues of agglomeration and leaching of active components. It also leverages the nanopores of COFs to construct unique confined microenvironments. This method offers an ideal platform for the construction of highly stable sensing interfaces.

The self-assembly strategy can increase the concentration of local active sites on the COFs surface, enhance catalytic ability and mass transfer efficiency, and improve the overall stability of the COFs [77]. The cost-effectiveness of self-assembly is derived from its use of thermal motion for generating microstructures without external costs. The tunable activities of COFs and nanomaterials offer adjustable driving forces for self-assembly, and these forces are utilized to design functional COFs electrochemical sensing microstructures. A revolutionary aspect of the self-assembly strategy is its achievement of ordered functional organization at the molecular level. This strategy directly incorporates functional groups as building blocks for skeleton construction, ensuring the long-range, periodic ordering of functional sites in the final material. A high degree of integration between crystallinity, porosity, and functionality is thus guaranteed. This integration enables the development of intrinsically functional materials that hold well-defined structure-property relationships and exceptional performance.

In practical applications, the functionalization strategies mentioned above can be utilized synergistically. Such an approach offers deeper insights into the application of functionalized COFs materials in electrochemical analysis.

### Reaction mechanism of COFs applied in electrochemical sensors

An electrochemical reaction is the basis for the working principle of an electrochemical sensor. The sensor identifies changes in electrical signals through the measurement of an analyte’s electrical properties or through the study of interfacial charge transfer at atomic and molecular scales [78]. COF-based functionalized materials can act as specific response sites for targeted analytes. The analytes then induce significant electrical signal changes through different mechanisms. A thorough understanding of the detection mechanism is therefore vital for designing and constructing high-performance electrochemical sensors based on COFs (Fig. 2C).

The most common detection mechanism for COF-based electrochemical sensors is charge transfer. An interaction between the analyte and the COFs causes this charge transfer, which affects the electrical signal’s intensity. The electrical signal intensity can decrease if charge from the COFs is transferred to the analyte; whereas, it increases in the opposite scenario [79]. It should be acknowledged that the sensing performance replies on the match between the COFs pore size and the analyte size. The porous structure of COFs displays different charge transfer paths. Charge can transfer directly to the analyte; alternatively, it can be conducted through ion migration inside the pores. When the size of an analyte matches the COF pore size, it can enter the pores smoothly. This offers an efficient transport path for ions and accelerates charge transfer. The contact probability between the analyte and the pore wall can also be enhanced through the domain-limiting effect that promotes specific interaction between the analyte and functional groups; moreover, it can improve the target recognition efficiency; whereas, a pore size that is too small can restrict the ion diffusion rate and slow the charge transfer process [80]. The analyte and COFs can combine through electrostatic attraction or a charge transfer effect if the analyte has an opposite charge to the COF’s functional groups or a polarizable electron cloud. This combination adsorbs the analyte and changes the electron cloud distribution of the COFs, which leads to a change in conductivity that is then converted into a detectable electrical signal. External environmental factors (such as temperature and pH) can also influence the protonation state of COFs functional groups, the analyte’s morphology, and the rate of molecular thermal motion, thereby regulating the charge transfer and adsorption kinetic processes [81].

Chemical transformations between COFs and an analyte, such as redox reactions, electrocatalytic reactions, condensation reactions, and enzyme catalysis, can also generate electrical signal changes. This process constitutes another detection mechanism for COF-based electrochemical sensors [82]. Doping strategies play a crucial role in this mechanism whereby the precise introduction of heteroatoms or metal centers into the COFs skeleton constructs highly active sites that efficiently catalyze target reactions. Through this process, COFs are transformed from mere conductive platforms into signal-amplifying interfaces to enhance sensing performance. The collapse of the crystal structure presents a final detection mechanism. An analyte-induced collapse of the COFs crystal structure inevitably changes the electrical signal intensity of the sensor. However, this destruction of the crystal structure affects the recycling of the sensor [83].

**Fig. 3:**
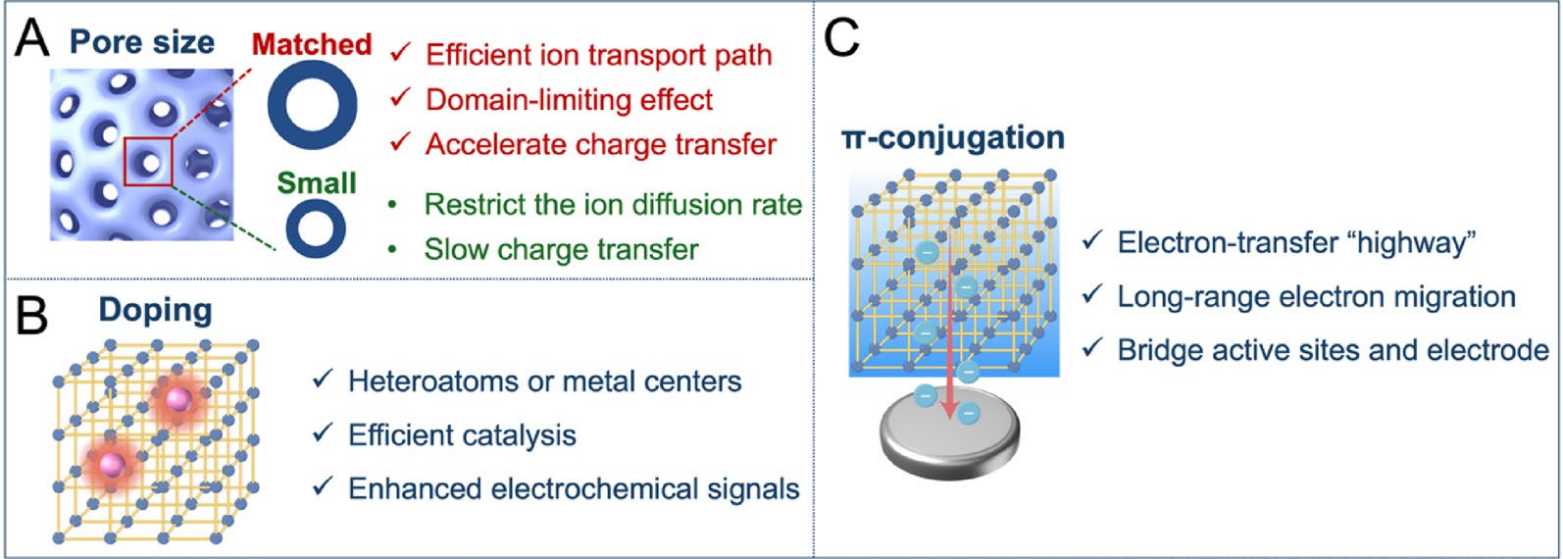
Schematic illustration of the electrochemical signal amplification mechanisms of COFs. (**A**) The well-defined nanopores facilitate ion diffusion and charge transfer. (**B**) Doping creates catalytic active sites for efficient electrocatalytic reactions. (**C**) The extended π-conjugated system shuttles electrons from active sites to the electrode surface with minimal resistance. Created with Figdraw

COFs represent excellent materials for constructing electrochemical sensors due to their strong physicochemical properties and diverse functionalization methods. Excellent electron conduction ability can be determined from the highly ordered π-π conjugated system and freely accessible regular pores inside COFs. The large specific surface area and high porosity of COFs offer an easily accessible surface with abundant molecular binding sites, which enhances the detection sensitivity. Doping strategies allow for precisely creating catalytic sites on the conjugated COFs skeleton, facilitating efficient electrocatalysis and introducing specific recognition capabilities via non-covalent interactions, thus enabling highly sensitive and precise detection of targets. Moreover, high crystallinity ensures structural stability, contributing to the long-term stability and repeated use of the sensor. The mechanisms of electrochemical signal amplification based on COF structures are illustrated in Fig. 3. The following sections specifically introduces the applications of COFs in cancer-related electrochemical sensors.

## Applications of COF-based electrochemical sensors in liquid biopsy

Tumor markers (including proteins, molecules, nucleic acids, cells, vesicles, etc.) offer crucial information regarding the course of cancer; therefore, monitoring their concentration in peripheral blood is crucial [17]. Traditional laboratory methods are insufficient for the required sensitivity and specificity for detection, a limitation attributed to the low abundance and varying clinical significance of tumor markers in peripheral blood [27]. New avenues for efficient liquid biopsy in cancer have been opened by electrochemical sensors that utilize COF-based novel functionalized materials and innovative reaction strategies. These sensors facilitate the early diagnosis and dynamic monitoring of the disease, thus advancing the goal of accuracy medicine. A comparative analysis of the performance across electrochemical platforms employing different strategies is summarized in Table 1.

**Table 1:** Comparative analysis of electrochemical platforms with different strategies

Sensing platform	Sensing mechanism	Advantages	Challenges	Prospects for clinical translation
Enzyme or enzyme-like catalytic amplification methods	Generating electroactive products via enzyme-catalyzed reactions	Significant amplification; well-established technology	Strict storage requirements; batch-to-batch variability	Relatively mature; limited by storage stability
Electrocatalytic reactions	Enhancing electron transfer utilizing electrocatalysts	Direct signal amplification; fast response; good reproducibility	Complex synthesis of catalysts; limited selectivity	Primarily at the research stage
DNA strategies	Achieving signal amplification through programmable DNA hybridization/assembly	High amplification efficiency; design flexibility; excellent stability	Complex probe design; stringent reaction conditions	Rapidly developing; hold immense potential for precise diagnosis and portable devices
Magnetic separation technologies	Separating and enriching target using magnetic nanoparticles	Enhanced signal-to-noise ratio; reduced matrix effect	Potential electrode interference; additional separation required	Highly mature; one of the most promising for clinical assays
Nano-functionalization technologies	Improving electrode performance with nanomaterials	Improved sensitivity; multi-functional integration; good stability	Limited reproducibility; potential toxicity concerns	Rapidly advancing; require standardized production and safety validation

### Lung cancer

Globally, lung cancer ranks as the most common cancer and represents the leading cause of cancer-related deaths. Lung cancer is categorized into two primary subtypes: non-small cell lung cancer (NSCLC), which constitutes approximately 80–85% of cases, and small cell lung cancer (SCLC) at 15–20%. The SCLC subtype is defined by high malignancy, a tendency for metastasis, and a poor prognosis [84]. Clinical practice currently employs several common tumor markers for lung cancer, including cytokeratin 19 fragment (CYFRA21-1), neuro specific enolase (NSE), ctDNA, and exosomes.

#### NSE and CYFRA21-1

Tumor markers vary among different subtypes of lung cancer. For instance, NSE is an acidic protease from neurons and neuroendocrine cells as a reliable marker for the diagnosis, monitoring, and prognosis of SCLC [85]. Hou et al. developed a method for the efficient electrochemical detection of NSE. Their process involved an in situ growth of COF_TP−TA_ on the surface of MIL-68-NH₂ through a Schiff base reaction. Then, electrostatic adsorption of CdS QDs formed a MIL-68-NH₂@COF_TP−TA_@CdS network heterojunction. The detection mechanism relied on NSE-dependent Cu²⁺ release from CuO acid, which chemically converted CdS in the heterojunction and allowed for highly sensitive NSE detection with a limit of detection (LOD) of 0.033 pg mL^− 1^ [86]. Creating COFs that possess both electrochemical and catalytic properties allows for the dual enhancement of catalytic activity and electrical signals. In another approach, Liang et al. constructed an electrochemical sensor for the electrocatalytic reduction of NSE by first modifying electrodes with vinyl-rich COFDva-TAB. For the signal probes, they combined COFs rich in iron porphyrin rings (COFp-Fepor NH₂-BPA) with gold nanoparticles (AuNPs) and conjugated antibodies. Dual signal amplification in an O₂-saturated environment was achieved as the iron porphyrin unit was center facilitated the Fe²⁺/Fe³⁺ redox reaction and also exhibited strong O₂ reduction catalytic activity. A wide detection range (500 fg mL^− 1^ to 100 ng mL^− 1^) and high sensitivity (8.82 J/lg (cNSE/ng mL⁻¹)) were characteristic of this electrochemical sensor [87]. For the detection of NSCLC, CYFRA21-1 is regarded as a tumor marker of primary importance [85]. To detect this marker, Cheng et al. constructed a sandwich-type electrochemical immunosensor. They created signal tags (TB-Au-COFs) by utilizing toluidine blue (TB) to modify AuNPs doped with COF polymers, which achieved additional signal amplification through electrocatalytic reduction. The sensor’s substrate enhancer and antibody carrier consisted of AuNPs-modified Ti₃C₂Tx-MXene (Au-Ti₃C₂Tx). The resulting immunosensor demonstrated high sensitivity for CYFRA21-1 at 0.1 pg mL^− 1^, along with a current response (0.5–1.0 × 10⁴ pg mL^− 1^) [88].

#### Secreted protein acidic and rich in cysteine (SPARC)

SPARC, a matrix cell glycoprotein that participates in processes such as cell adhesion, proliferation, differentiation, and extracellular matrix degradation, has emerged as a new lung cancer marker. Its status is supported by studies indicating its overexpression in tumor stroma and its association with poor prognosis in NSCLC [89]. A polymer-assisted surface modification strategy was utilized by Lu et al. to functionalize COFs. The polymer increased the number of active groups on the COFs surface, a modification that enabled the dense attachment of peptides and conferred excellent protein targeting properties. The same mechanism was employed to modify horseradish peroxidase (HRP) onto the COFs. Several properties of COFs contributed to enhanced signal intensity; for instance, the large specific surface area permitted a large amount of HRP to bind flexibly. In addition, the material’s porosity and excellent biocompatibility offered more molecular binding sites and promoted higher activity. The prepared functionalized COFs (PHCs) were therefore suitable for the quantitative detection of SPARC [90].

#### CtDNA

ctDNA molecules are double-stranded DNA fragments, 90 to 320 nucleotides in length, that are derived from tumor cells. These fragments carry cancer-specific mutations, making them important tumor markers for liquid biopsy [91]. In NSCLC patients, both EGFR T790M and EGFR L858R ctDNA demonstrate high sensitivity and specificity. Their detection is therefore crucial for understanding NSCLC occurrence, guiding targeted therapy, and determining prognosis [92].

**Fig. 4:**
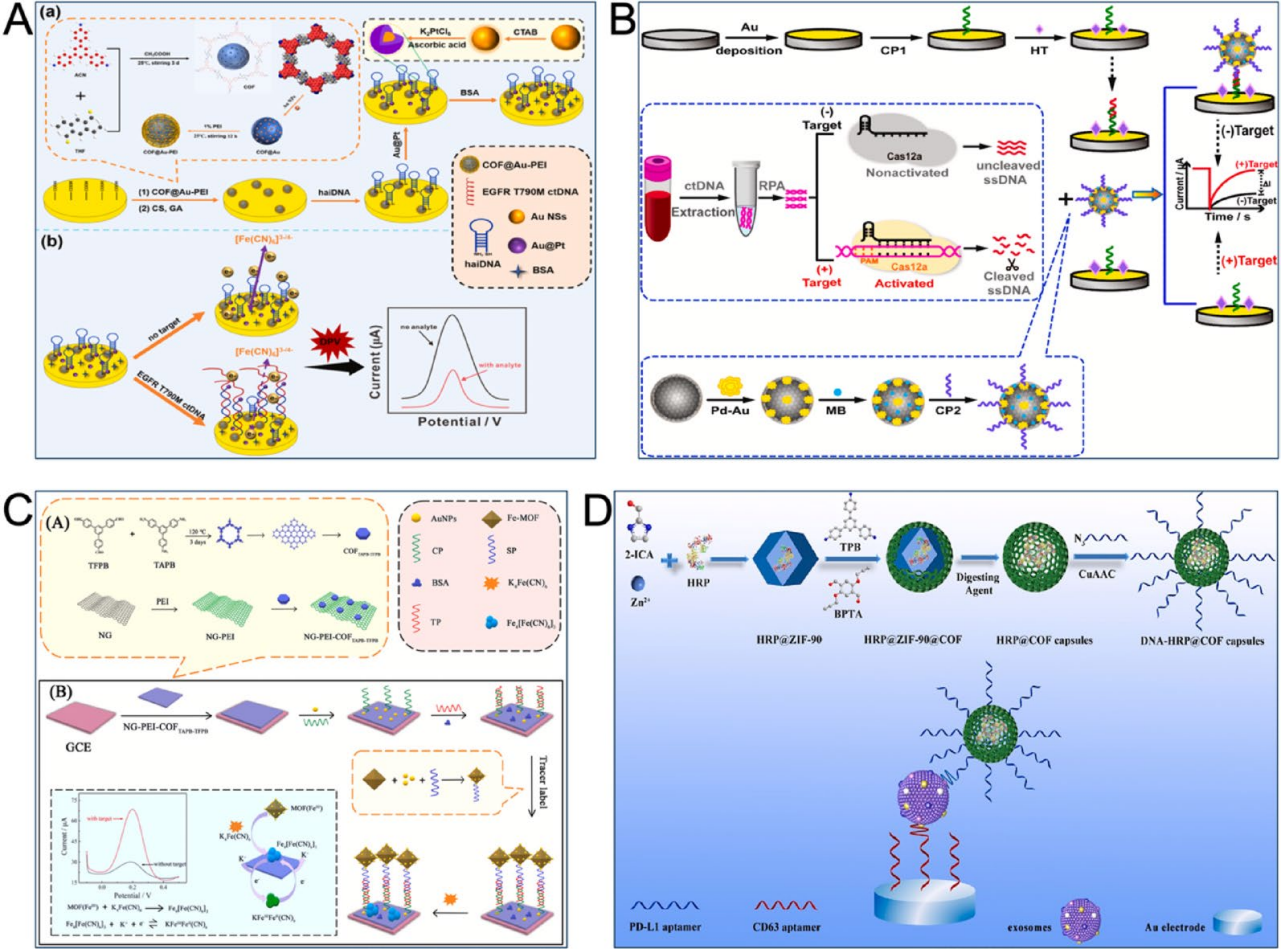
COF-based electrochemical sensors for the detection of lung cancer markers. (**A**) Diagrammatic sketch of an interference-based EGFR T790M ctDNA sensor with COF@Au-PEI and the haiDNA probes [93]. Copyright 2025, Elsevier. (**B**) Schematic representation of EGFR L858R ctDNA sensor composed of CRISPR/Cas12a system and Fe₃O₄@COF/PdAu [94]. Copyright 2022, Elsevier. (**C**) Illustration of EGFR L858R ctDNA sensor integrated NG-PEI-COFTAPB-TFPB with electrochemical sandwich structure [95]. Copyright 2022, Elsevier. (**D**) Schematic diagram of DNA-functionalized COF capsules for electrochemical analysis of PD-L1^+^ exosomes [96]. Copyright 2023, Elsevier

Li et al. developed an interference-based COF electrochemical sensor to detect EGFR T790M ctDNA in plasma. Their design utilized polyethyleneimine-coated AuNPs-modified COFs (COF@Au-PEI) as optimized conductive particles for the working electrode (WE). This approach accelerated electron transfer and improved the WE’s structure. A specific hairpin DNA probe (haiDNA) targeting EGFR T790M ctDNA and Au@platinum (Au@Pt) nanohybrids were then utilized to modify the WE. When the target bound to haiDNA, it interfered with the electrochemical reaction, causing a proportional decrease in the current signal. The sensor’s complex multilayer design yielded a good LOD of 0.6 pM and high selectivity, indicating no signal change for the wild-type allele that contained only a single base pair difference (Fig. 4A) [93].

A different approach by Liu et al. also combined DNA strategies, adeptly leveraging the enrichment and sensing capabilities of COFs to detect low-concentration EGFR L858R ctDNA. Their method involved conjugating COFs with PdAu for signal amplification and utilizing methylene blue (MB) as the signal unit. To achieve multi-level signal amplification, they anchored the nucleic acid probe CP2 to Fe₃O₄@COF/PdAu through Au-S bonds. A highly specific and sensitive electrochemical sensor was constructed by utilizing the accurate targeting capability of the CRISPR/Cas12a system. The trans-cleavage activity of Cas12a was silenced without the target, which leaved single-stranded DNA intact to produce a high signal. In the presence of the target, however, Cas12a’s trans-cleavage activity was activated to cleave non-specific single-stranded DNA. This cleavage prevented CP1, fixed on the electrode, from connecting to CP2 through single-stranded DNA, leading to a decrease in signal. This process enabled the quantitative detection of EGFR L858R ctDNA based on current changes. This electrochemical sensor achieved an LOD of 3.3 aM, with detection accuracy confirmed by ddPCR and sequencing (Fig. 4B) [94].

In another study, nitrogen-doped graphene (NG) nanocomposites functionalized with PEI and COF_TAPB−TFPB_ (NG-PEI-COF_TAPB−TFPB_) were first utilized as a sensing substrate. The material’s porous structure, excellent conductivity, and dispersibility promoted electron transfer, offered large specific surface areas and multiple active sites. Iron-based MOFs (Fe-MOFs) modified with AuNPs were also employed as tracer labels. A reaction between FeIII in the Fe-MOF and K₄[Fe(CN)₆] generated electroactive prussian blue (PB), resulting in significantly amplified electrochemical detection signals. This amplification offered quantitative evidence for the sensitive detection of EGFR L858R ctDNA with a wide linear range (100 fM–100 nM) and an LOD of 7.65 fM (Fig. 4C) [95]. Alternatively, P-COF-AuNPs (PEI and AuNPs-functionalized COFs) could act as interface materials. Their use enhanced the sensing interface’s effective area and conductivity, increased the solid-phase loading of capture probes, and finally improved the EGFR L858R ctDNA sensor’s sensitivity [97].

#### Exosomes

Exosomes are EVs produced by the endosomal system in the cytoplasm, with a size range of 30 to 150 nm. Various tumor cells release these highly heterogeneous vesicles, which participate in forming the TME. The reprogramming and phenotypic conversion of various cells in the TME can be promoted by bioactive molecules on exosome surfaces, a process that drives tumor progression and distant metastasis [13]. PD-L1 on the surface of exosomes plays a key role in cancer progression. Studies have demonstrated that PD-L1^+^ exosomes exhibit immune-suppressive effects. They also reflect the tumor’s immune status and exhibit a good correlation with immunotherapy responsiveness [98].

Wang et al. first utilized 2D ultra-thin COF nanosheets (2D COF NSs) for the electrochemical analysis of PD-L1^+^ exosomes. They synthesized COF-367 nanosheets with a 2D high π-conjugated nanostructure. AuNPs were then grown in situ in this porous structure to enhance charge mobility and the sensing layer’s response speed, while also enabling the efficient immobilization of DNA probes. The CRISPR/Cas12a system was also introduced. The presence of the target caused the activator DNA complementary to the aptamer to dissociate from the MB-aptamer DNA complex. This event activated the trans-cleavage activity of CRISPR/Cas12a. The system then specifically cleaved the MB-aptamer DNA, which achieved ultra-sensitive detection of PD-L1^+^ exosomes and verified the method’s feasibility for NSCLC diagnosis [99].

To ensure the effective occurrence of enzymatic catalytic reactions, Lin et al. encapsulated HRP in biodegradable ZIF-90. This material also represented a template for COFs shell growth, which was synthesized under mild conditions to enhance HRP conformational freedom and improve mass transfer efficiency. Cu(I)-catalyzed azide/alkyne cycloaddition (CuAAC) was then utilized to modify azide-labelled DNA onto COFs containing alkyne groups. This step prepared DNA-functionalized COF capsules (DNA-HRP@COF) for the electrochemical sensing analysis of PD-L1^+^ exosomes. The ordered arrangement of DNA chains on the COFs surface significantly improved binding efficiency. Meanwhile, the abundant HRP inside achieved signal amplification. This approach finally achieved an LOD as low as 87 particles µL^− 1^, effectively distinguishing exosomes derived from NSCLC (Fig. 4D) [96].

### Breast cancer

As the most common cancer in women, breast cancer has the highest incidence and mortality rates. Reducing mortality and improving survival rates among breast cancer patients are dependent on the crucial measures of early detection and timely treatment [1]. Numerous breast cancer markers exist. These include the clinically most commonly utilized CEA and CA15-3, as well as emerging biomarkers such as human epidermal growth factor receptor 2 (HER2), PD-L1, CTCs, and exosomes.

#### Protein biomarkers

**Fig. 5:**
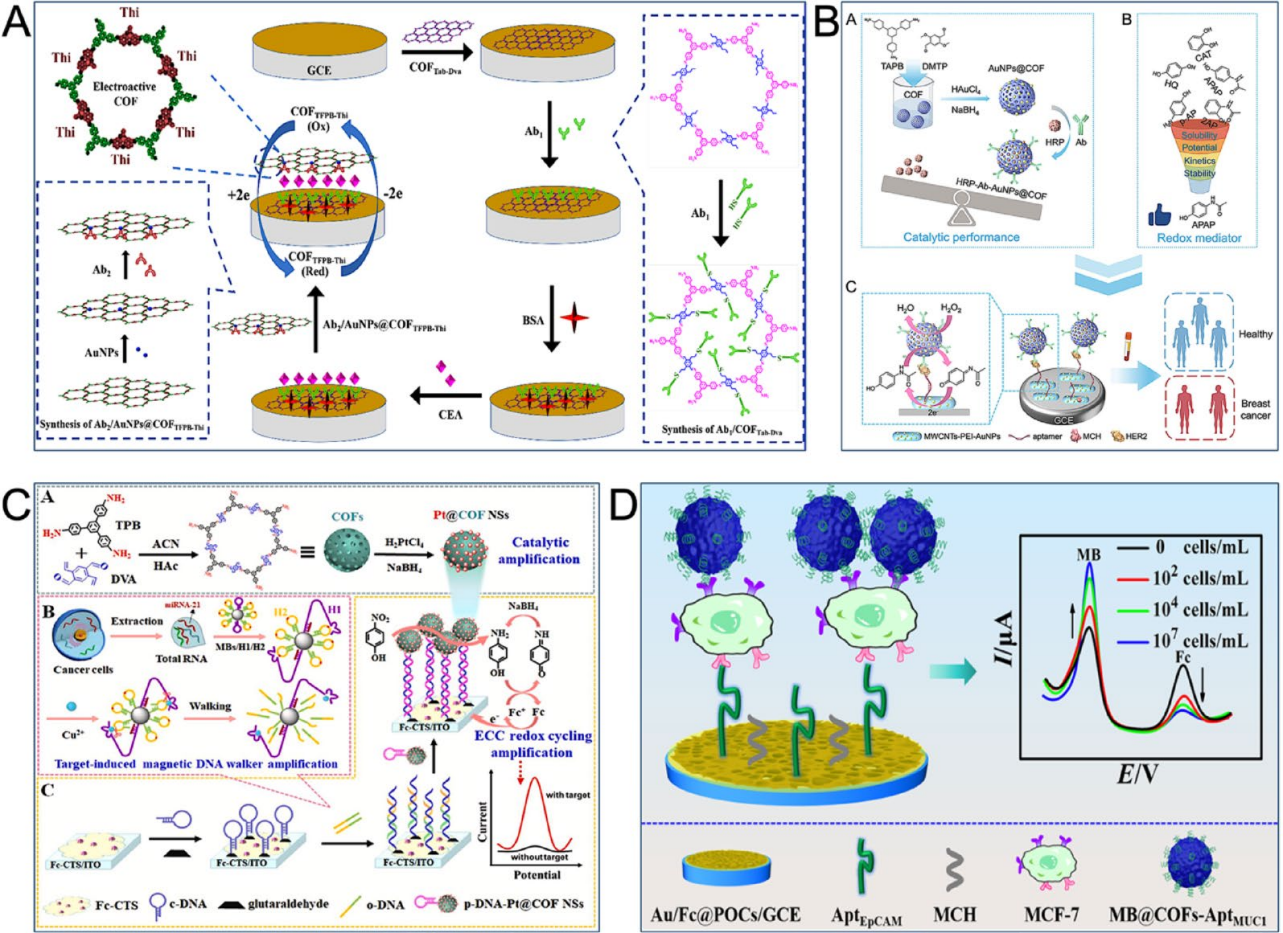
COF-based electrochemical sensors for the detection of breast cancer markers. (**A**) Diagram of CEA sensor using electroactive 2D COFs as signal probes [100]. Copyright 2022, American Chemical Society. (**B**) Schematic diagram of HER2 sensor based on 3D mesoporous COFs-immobilized HRP and the novel redox medium APAP [103]. Copyright 2024, Springer Nature. (**C**) Schematic illustration of miRNA-21 sensor utilizing Pt@COF NSs-based ECC redox cycling [113]. Copyright 2023, Elsevier. (**D**) Illustration of a dual recognition strategy-based sensor with MB@COFs as the label materials for CTCs detection [114]. Copyright 2024, American Chemical Society

Clinical testing methods for the traditional breast cancer markers CEA and CA15-3 have several drawbacks, including time-consuming processes and high costs [26]. To address this, Liang et al. constructed a sandwich-type electrochemical immunosensor for CEA detection by utilizing two 2D COFs with abundant active sites. Their design involved conjugating vinyl-functionalized COFTab-Dva with Ab1 through a thiol-alkene ‘click’ reaction. A signal probe was created by loading Ab2 onto AuNPs-modified electroactive COFTFPB-Thi through Au-S bonds. This sensor enabled quantitative detection and signal amplification of CEA, achieving a low LOD of 0.034 ng mL^− 1^ (Fig. 5A) [100]. In a different approach, Dezhakam et al. developed a sensor for CA15-3 detection by coating MOFs MIL-156 with crystalline COFs to form core-shell structured MIL-156 MOF@COF nanocomposites. These were electrodeposited with AuNPs onto an electrode surface, creating a highly sensitive electrochemical sensor. The resulting device demonstrated a linear range of 30–100 nU mL^− 1^ and could distinguish between breast cancer patients and healthy individuals with high confidence [101].

The US Food and Drug Administration (FDA) has approved the transmembrane protein HER2 as one of the few reliable breast cancer biomarkers [102]. The use of enzyme-catalyzed reaction strategies can enhance sensor sensitivity, while the enzyme’s specificity also ensures selectivity. Our research group developed an enzyme-enhanced electrochemical sensing platform for HER2 detection that involved immobilizing HRP on 3D mesoporous COFs and utilizing 4-acetamidophenol (APAP) as a novel redox medium. The functionalized COFs (HRP-Ab-AuNPs@COF) was a carrier for HRP, exhibiting superior catalytic activity and electrochemical performance compared to free HRP while achieving target recognition and signal amplification. APAP was selected over various HRP candidate media to replace traditional medium hydroquinone, which resulted in a high electrochemical signal-to-noise ratio. In combination with functionalized multi-walled carbon nanotubes (MWCNTs), the electrochemical sensor achieved a low LOD of 0.418 pg mL^− 1^. This performance allowed for high-precision differentiation between benign and malignant breast diseases and patients at different cancer stages with greater accuracy and reliability than CEA and CA15-3 (Fig. 5B) [103]. Considering the crucial role of soluble PD-L1 (sPD-L1) in breast cancer, we also developed an enzyme-catalyzed electrochemical aptasensor for its non-invasive detection of sPD-L1 in peripheral blood. We designed an enzyme-catalyzed signal probe based on COFs (COFs-AuNPs-Ab-HRP) and utilized PEI-functionalized MWCNTs as the substrate material, applying this system for the first time to sPD-L1 detection. This aptasensor performed excellently across multiple applications, including cell culture supernatants and human peripheral blood, and demonstrated higher sensitivity (0.143 pg mL^− 1^) than commercialized sPD-L1 ELISA kits [104].

Vascular endothelial growth factor (VEGF), which is associated with various diseases, forms four subtypes through alternative exon splicing. The VEGF165 subtype is overexpressed in breast cancer cells and is generated when the exon encoding exon 6 is absent [105]. To detect VEGF165, Cui et al. synthesized porphyrin-based COFs (p-COF) utilizing 5,10,15,20-tetra(4-aminophenyl)porphyrin and 1,10-phenanthroline-2,9-dicarboxaldehyde. The p-COF’s abundant C-N functional groups and π-stacking planar nanostructure were leveraged to enhance sensor performance [106]. Another potential biomarker, the cell surface transmembrane glycoprotein CD44, plays a key role in breast cancer onset, progression, and metastasis [107]. A sensing platform for its detection was constructed utilizing carbon ionic liquid electrode (CILE) based on custom templates. An enzyme-free signal amplification strategy employed a thiourea/Au/COFs (THI/Au/COF) signal probe, which introduced hydrogen peroxide (H₂O₂) and phosphotungstic acid (PW12) with peroxidase (POD)-like activity. The final sandwich-type electrochemical sensor for CD44 exhibited a linear detection range of six orders of magnitude with an LOD as low as 0.71 pg mL^− 1^, rendering it highly valuable for early diagnosis and screening of breast cancer in low-income countries [108].

#### Nucleic acid biomarkers

MiRNAs are short non-coding ribonucleic acid molecules (20–25 nucleotides) found in eukaryotic cells. Their abnormal expression is associated with various diseases, including cancer and cardiovascular disease [109]. For instance, research indicated that miRNA-21 promoted tumor growth and metastasis, and its levels in the serum and saliva of breast cancer patients were closely associated with disease progression [110]. Therefore, efficient detection of miRNA-21 is considered an effective means for breast cancer diagnosis. Feng et al. developed an electrochemical sensor for this purpose, which was based on ferrocene (Fc)-functionalized COFs nanoprobes and a targeted catalytic hairpin assembly (CHA) strategy. By introducing the electroactive molecule Fc and DNA complementary probes onto the COFs, they synthesized a novel COFs electrochemical active probe with good biocompatibility, stability, and electrochemical performance. The electrical signal was then further amplified by combining a tetrahedral DNA nanostructure (TDN) sensing interface with the CHA amplification strategy. This sensor exhibited a wide linear response range for miRNA-21 from 1 fM to 10 nM with an LOD of 0.33 fM, and it demonstrated acceptable accuracy and precision in actual sample analysis [111]. In a similar approach, Wang et al. also utilized the CHA signal amplification strategy. They designed an electrochemical sensor based on lanthanide Eu³⁺-functionalized COFs (Eu@TAPP-COF), utilizing 1,3,5-benzenetricarboxaldehyde and 20-tetra(4-aminophenyl) porphyrin as raw materials. This sensor offered stable and efficient detection of miRNA-21, indicating a linear response range of 100 aM to 100 pM and an LOD of 21 aM, offering new opportunities for the early diagnosis and prognosis assessment of breast cancer [112].

For miRNA-21 detection, a separate, highly sensitive, enzyme-free electrochemical sensor was also designed utilizing porous COFs as carrier. To prepare Pt@COF nanospheres (Pt@COF NSs), Pt was reduced in situ in the COFs nanopore structure. This method prevented Pt particle migration while imparting ideal stability and catalytic activity to the Pt@COF NSs. This enabled the construction of an electrochemical-chemical-chemical (ECC) redox cycle to achieve signal enhancement. Cumulative signal amplification was also achieved through the orderly induction of target-induced magnetic DNAase walkers. This method achieved an LOD as low as 47.5 aM and demonstrated reliable detection capability and interference resistance in serum and cell samples (Fig. 5C) [113].

ctDNA represents an economical and feasible tumor marker. Studies have demonstrated PIK3CA mutations to be crucial factors in breast cancer progression. Since E545 is the most common mutation site in the PIK3CA gene, the PIK3CA E545K ctDNA has become a key biomarker for breast cancer [115]. An electrochemical sensor was designed for detecting PIK3CA E545K ctDNA by combining a CRISPR/Cas9n-driven 3D DNA walker with gold-nanosphere-like COFs (COFs-AuNPs). The DNA walker probe was activated only in the presence of the target, binding to the support probe to form a double strand. The Cas9n/sgRNA complex then specifically cleaved this strand, which generated a large number of DNA fragments to amplify the signal. COFs-AuNPs facilitated electron transfer and increased the number of biomolecular binding sites. The sensor’s specificity was enhanced by Cas9n, and it achieved an LOD of 1.76 aM, demonstrating potential for early breast cancer diagnosis [116].

#### Cell and vesicle biomarkers

In addition to soluble biomarkers, insoluble biomarkers include CTCs and EVs. CTCs are malignant cells that enter the bloodstream after detaching from primary or metastatic tumors and carry key information from the primary tumor site. Their ability to invade distant tissues leads to cancer-related deaths [23]. The detection of CTCs is therefore crucial for improving breast cancer survival rates, as metastasis is the cause of most related deaths.

The low abundance of CTCs in peripheral blood presents a challenge. To address this, Tian et al. designed a dual-signal electrochemical sensor, utilizing a substrate of gold-film-modified porous organic cages loaded with Fc (Au/Fc@POCs) and MB-encapsulated COFs (MB@COFs) as the marker materials. Upon the capture of breast cancer cells, the Fc electrochemical signal decreased while an amplified electrochemical signal from MB was generated. This switch-mode signal effectively eliminated background interference, amplified the detection signal, and enabled accurate CTCs detection with an LOD as low as 1 cell mL^− 1^. The method was successfully applied in the high-precision measurement of CTCs in clinical samples (Fig. 5D) [114]. Guo et al. confronted the issue of CTCs phenotypic heterogeneity caused by epithelial-mesenchymal transition (EMT) by employing a novel 3D multivalent aptamer recognition strategy (MARS). This approach overcame EMT heterogeneity and enhanced detection sensitivity. An exonuclease III (Exo III)-mediated DNA walker signal amplification strategy was employed to further enhance sensitivity. The sensor, combined with the superior performance of the nanomaterials (Au@COF-LZU1@Ru), detected CTCs in a range of 8 to 1 × 10⁵ cells mL^− 1^, with an LOD of 2 cells mL^− 1^. Actual sample analysis (AUC = 0.97) confirmed its potential for clinical application [117].

Exosomes from breast cancer cells are an ideal target for non-invasive breast cancer diagnosis because they contain valuable information from their parent cells. Wang et al. proposed a template-free, in situ synthesis of size-tunable spherical COF capsules for enzyme encapsulation (HRP@COF). In these capsules, the COFs cavity maintained high conformational freedom of HRP, which enhanced the enzyme’s activity and stability. Based on this material, an electrochemical sensor for detecting breast cancer-derived exosomes was constructed. The sensor utilized the encapsulated HRP to catalyze the oxidation of H₂O₂ to 3,3′,5,5′-tetramethylbenzidine (TMB). This reaction generated a strong electrochemical signal for quantitative exosome analysis without further amplification, with an LOD as low as 80 particles uL^− 1^ [118]. Zhang et al. designed a ternary nanohybrid (Ru-HAuTiO₂) utilizing hollow sea urchin-shaped titanium dioxide (HTiO₂) to achieve probe-free free exosome capture. They labeled CD63-specific aptamers with dopamine-modified COFs (PDA@COF). This process formed a Ru-HAuTiO₂/exosome/Apt-PDA@COF sandwich structure on the electrode, which enabled the sensitive quantification of exosomes and the phenotypic analysis of surface proteins [119].

### Ovarian cancer

The most lethal malignant tumor affecting the female reproductive tract is ovarian cancer. Early stages typically lack specific clinical symptoms. Since symptoms only appear when the disease is advanced, the prognosis is highly deficient, posing a serious threat to women’s health [84].

**Fig. 6:**
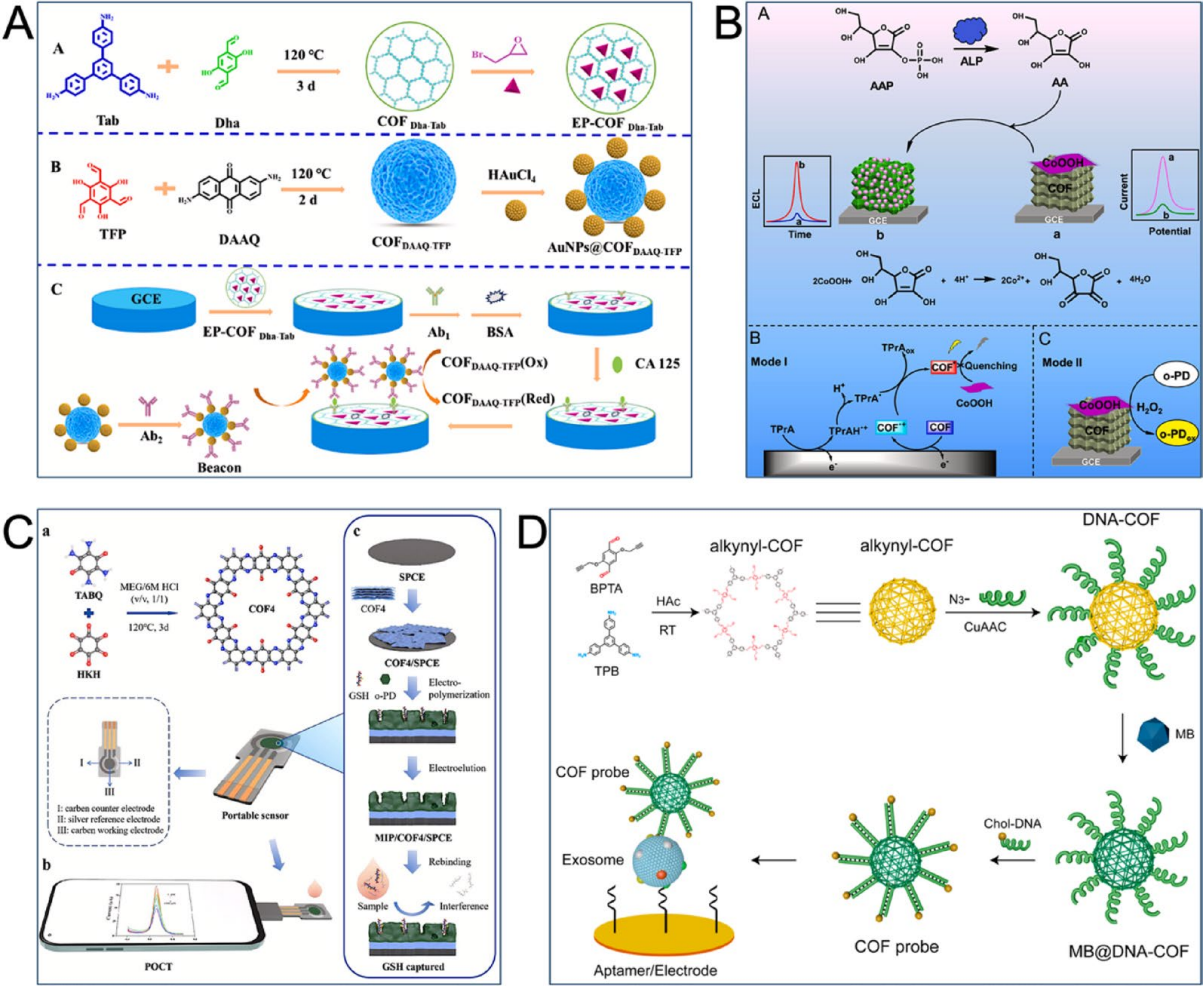
COF-based electrochemical sensors for the detection of ovarian cancer and cervical cancer markers. (**A**) Fabrication process of the electrochemical immunosensor for CA125 based on electroactive AuNPs@COF_DAAQ−TFP_ [121]. Copyright 2025, Elsevier. (**B**) Diagram of a dual-mode sensor for ECL and EC sensing of ALP integrated by fully π-conjugated COFs and CoOOH nanosheets [127]. Copyright 2023, Elsevier. (**C**) Schematic illustration of a portable imprinted electrochemical sensor with engineered conductive COFs for GSH detection [128]. Copyright 2025, Elsevier. (**D**) Schematic diagram of a DNA-COF-based electrochemical sensor for the detection of exosomes [129]. Copyright 2022, American Chemical Society

CA125 is the most common tumor marker for ovarian cancer, existing as a high-molecular-weight mucinous glycoprotein on the surface of the cancer cells [20]. The electrochemical analysis of CA125 significantly involves electroactive COFs. Jin et al. synthesized epoxy-functionalized COFs (EP-TD-COF) with a large specific surface area that exposed active sites and stably covalently bound Ab1. They also synthesized electropositive AuNPs@COFBTT-DGMH. This material enriched negatively charged signal probes [Fe(CN)₆]³/⁴, accelerating mass transfer and electron transport. In addition, it offered abundant binding sites for Ab2, which efficiently amplified electrochemical signals. These biocompatible 2D COFs effectively preserved the activity of biomolecules, offering a favorable microenvironment for their loading. The stability and sensitivity of the CA125 immunosensor (LOD as low as 0.089 mU mL^− 1^) were enhanced by their synergistic effects [120]. In other work, the research group utilized 2,6-diaminanthraquinone and 2,4,6-trifluorophenol to synthesize COF_DAAQ−TFP_, a microspherical electroactive copolymer with abundant pores and uniform morphology. They modified AuNPs as signal probes. A positive correlation existed between the signal response of AuNPs@COF_DAAQ−TFP_ and CA125 concentration. This achieved a linear range of 0.01 to 100 U mL^− 1^ and a low detection limit of 0.0067 U mL^− 1^ (Fig. 6A) [121]. An et al. also successfully integrated cerium-MOF (Ce-MOF) with toluene-based COF/carbon nanotubes (TPN-COF/CNT). Immobilization of antibodies and the formation of immune complexes with CA125 were facilitated by hydrogen bonds and π-π interactions between TPN-COF’s triazine rings and the tricarboxylic acid ligands. The electron transfer rate was enhanced by abundant -COOH groups in tricarboxylic acid and the unsaturated Ce³⁺ sites in the porous Ce-MOF. utilizing the hybrid material Ce-MOF/TPN-COF/CNT, a highly efficient electrochemical immunological platform was constructed, achieving a low CA125 detection limit (0.000088 U mL^− 1^) in a 0.0001 to 100 U mL^− 1^ concentration range [122].

Sialic acid (SA) is a specific sugar located at the terminal end of cell surface glycoproteins and glycolipids. SA levels correlate significantly with ovarian cancer incidence, according to multiple studies [123]. Therefore, SA can act as a novel biomarker reflecting TME changes at earlier stages. Researchers have designed various functionalized COFs for SA detection to enable earlier ovarian cancer diagnosis. Hui et al. synthesized vinyl-functionalized COFs (TMTA-COF) through the Knoevenagel condensation reaction, utilizing 2,4,6-trimethyl-1,3,5-triazine and p-benzaldehyde as raw materials. Then, a thiol ‘click’ reaction grafted 4-mercaptobenzoboronic acid onto TMTA-COF. Abundant reaction sites for grafting boronic acid were offered by the alkene bond. This resulted in novel boronic acid-functionalized COFs (TMTA-PBA-COF) capable of accurate SA detection [124]. Moreover, hydrogels prepared utilizing COFs TpPa-1 functionalized with dyes (TpPa-1@Dye) enabled ultra-sensitive detection of SA across a wide linear range (10^− 8^–10^− 2^ M), even when major serum components were present [125].

Alkaline phosphatase (ALP), a naturally occurring hydrolase catalyzing the dephosphorylation of phosphate substrates, is widely present in mammalian placenta, bones, and liver [126]. Serum ALP is considered an ovarian cancer marker. A dual-mode sensor for electrochemical luminescence (ECL) and electrochemical (EC) detection of ALP was constructed by Cui et al., based on fully π-conjugated COFs and cobalt oxyhydroxide (CoOOH) nanosheets. Fully π-conjugated COFs (TCPB-DMTA-COF) were formed utilizing 2,5-Dimethoxy-p-benzenediamine (DMTA) and 1,3,5-tri(4-cyanomethylphenyl)benzene (TCPB) through Knoevenagel polymerization reaction. These COFs served as highly efficient ECL luminescent materials. When ALP was absent, CoOOH reduced the TCPB-DMTA-COF signal through the RET mechanism. Meanwhile, it catalyzed the oxidation of o-phenylenediamine (o-PD) by H₂O₂ to generate a high DPV signal. When ALP was present, CoOOH hydrolysed L-ascorbic acid-2-phosphate (AAP), forming L-ascorbic acid (AA). AA reduced CoOOH to Co²⁺, leading to the decomposition of CoOOH nanosheets. This decomposition restored the ECL signal while reducing the DPV peak current. Compared to single-mode sensors, this dual-mode sensor offered higher reliability and accuracy by effectively eliminating various errors (Fig. 6B) [127].

### Cervical cancer

Cervical cancer incidence and mortality rates have significantly decreased in recent years. A counter-trend, however, is the diagnosis of new cases at a younger age [2]. The early detection of cervical precancerous lesions is therefore crucial for improving cure and survival rates.

The pan-cancer biomarker CEA finds application in diagnosing cervical cancer-related diseases. To detect trace amounts of CEA and HIV dual targets, Yan et al. developed a novel electrochemical sensor. The sensor was based on ordered 2D porous ultra-thin COF films and AgInS₂ quantum dots (QDs). Its sensitive and rapid detection effectively avoided false positives and negatives, which had important implications for the early diagnosis of HIV-induced invasive cervical cancer [130].

Glutathione (GSH) is a tripeptide widely distributed in tissues and cells that participates in several key processes. These functions include maintaining redox homeostasis, inhibiting reactive oxygen species (ROS), counteracting oxidative stress, and promoting tumor resistance [131]. The detection of GSH can therefore assess the TME status of cervical cancer. For this purpose, Haotian et al. synthesized engineered nitrogen-containing heterocyclic fused π-conjugated COFs with varying conductivities. They modified SPCEs with the most conductive 2D crystal COF4 (240% conductivity) to create an effective electron transport layer indicating excellent signal enhancement effects. This work enabled the construction of a portable molecularly imprinted electrochemical sensor for bedside GSH detection. The single pristine COF demonstrated superior detection performance (concentration range: 1–1000 µM; LOD: 0.191 µM) and improved selectivity against thiol interferents compared to the traditional AuNPs co-modification strategy (Fig. 6C) [128].

Covalent modification to functionalize COFs ensures their stability in complex media while minimizing negative effects. Lu et al. applied this concept to detect cervical cancer-derived exosomes, utilizing a mild and efficient CuAAC reaction to covalently modify COFs with DNA (DNA-COF). This method achieved efficient fixation of biomolecules and uniform distribution of DNA. They then developed MB@DNA-COF nanoprobes where DNA functionalization enabled exosome capture and a large amount of encapsulated MB amplified the signal, resulting in a highly sensitive electrochemical analysis (Fig. 6D) [129]. In a similar approach, Han et al. utilized DNA-COF by loading HRP and cholesterol-labelled DNA (HDCs) onto its surface through simple mixing. This loading process leveraged the COF’s large specific surface area, non-covalent HRP-COF interactions, and the Watson-Crick base complementary pairing principle. Since HDCs possessed dual functions for exosome-targeted recognition and signal amplification, the constructed electrochemical sensor exhibited excellent linear detection performance in the 10^4^–10^7^ particles µL^− 1^ range [132].

### Prostate cancer

**Fig. 7:**
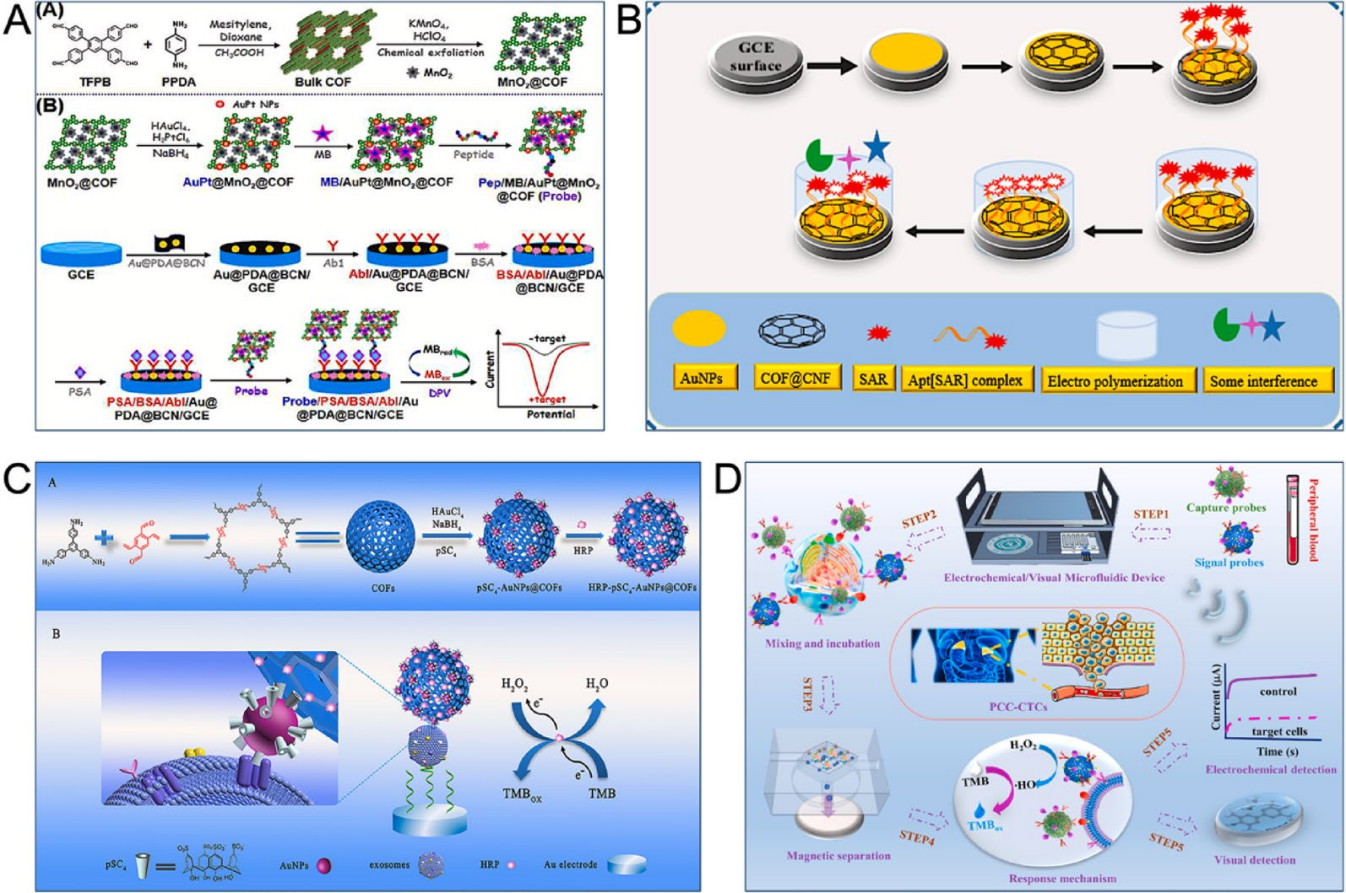
COF-based electrochemical sensors for the detection of prostate cancer, CRC and PCC markers. (**A**) Illustration of the affinity peptide-antibody sandwich electrochemical assay for PSA based on MnO_2_-functionalized COFs [133]. Copyright 2021, Elsevier. (**B**) Schematic diagram of SAR sensor combining MIP and aptamer strategies [137]. Copyright 2025, Elsevier (**C**) Schematic illustration of CRC-derived exosomes electrochemical detection based on HRP-pSC4-AuNPs@COFs nanoprobe [141]. Copyright 2020, Elsevier. (**D**) Schematic diagram of electrochemical/visual microfluidic detection with COF@Pt nanozyme-based device [142]. Copyright 2022, Elsevier

PSA is a primary biomarker for prostate cancer, which is a major global public health issue and has the second-highest incidence rate among men, after lung cancer [21]. For PSA detection, Zheng et al. proposed a new strategy that utilized manganese dioxide (MnO₂) functionalized COFs to amplify signals in an affinity peptide-antibody sandwich electrochemical assay. Their approach involved several key steps. First, they synthesized poly(dopamine)-coated boron-doped carbon nitride (Au@PDA@BCN) as a sensing platform for immobilizing a large amount of Ab1. Meanwhile, gold-platinum nanoparticles with MnO₂-modified COFs (AuPt@MnO₂@COF) were prepared to function as a nanocatalyst and offer ordered nanopores for MB enrichment and amplification. These nanoparticles were then combined with PSA affinity peptides to prepare Pep/MB/AuPt@MnO₂@COF nanoprobes. The constructed sandwich electrochemical sensor measured the redox signals of MB in the presence of PSA and achieved an LOD as low as 16.7 fg mL^− 1^. Moreover, the sensor demonstrated ideal selectivity and stability for PSA detection in real samples (Fig. 7A) [133]. In a different approach, Zhou et al. designed a magnetic COF-encapsulated osmium (Os) nanocluster enzyme (Fe₃O₄@COF@Os) that exhibited POD specificity for PSA sensing. The catalytic activity and specificity of Fe₃O₄@COF@Os could be modulated by the functional groups of the deoxygenation agent and COF ligands, a feature which enabled interference-free, ultra-sensitive PSA detection. This method’s clinical sample testing achieved 100% accuracy and a correlation coefficient of 0.998 when compared to commercial ELISA kits, confirming its suitability for rapid and accurate POCT [134].

Serine (SAR), an endogenous and non-protein-derived metabolite of glycine, is another critical biomarker as its concentration increases significantly during prostate cancer progression [135]. To detect SAR, Hamdi et al. first synthesized hexagonal 2D imine-based COFs by utilizing triangular building blocks with three melamine groups and linear linkers for the first time. The resulting COFs featured a highly porous structure with a high specific surface area, which offered higher binding capacity, efficient adsorption, and structural stability. A significant increase in the electrode surface area resulted from the combination of hydrogen bonding interactions between aptamers and COFs with the high porosity of the COFs. The prepared SAR sensor exhibited two linear ranges (0.5–700 fM and 10 pM–0.12 nM) with a detection limit of 0.15 fM [136]. The same research group also developed a separate electrochemical sensor based on molecularly imprinted polymers (MIPs) and aptamer strategies. In this sensor, AuNPs and COF@carbon nanofibres (COF@CNF) acted as organized nanocomposites to stabilize aptamer, while the electropolymerization of PDA around the Apt/COF@CNF/AuNPs enhanced selectivity. The synergistic action of MIPs and the aptamer captured SAR. Utilizing electrochemical impedance spectroscopy (EIS) for quantification, the sensor was measured with linear ranges of 500 fM–50 pM and 50–350 pM, and a detection limit of 0.166 pM, respectively. Its performance was then appropriately evaluated in urine samples (Fig. 7B) [137].

### Hepatocellular carcinoma (HCC)

Alpha-fetoprotein (AFP), a salicylate-modified glycoprotein with an approximate molecular weight of 68 kD, is a key biomarker for HCC. The yolk sac and liver produce AFP during early embryonic development [27]. For their electrode platforms, Bölükbaşi et al. utilized Fe₃O₄NPs@COF/AuNPs and SiO₂@TiO₂-based double-coated magnetic nanoparticles (MNPs@SiO₂@TiO₂). They respectively coupled AFP capture and detection antibodies through Au-NH₂ and electrostatic/ion interactions, a method that achieved target capture and signal amplification to an LOD of 3.30 fg mL^− 1^. The proposed AFP electrochemical immunosensor was therefore a promising tool for HCC diagnosis [138].

Cholate glycine (CG) is a novel specific biomarker for HCC, as it is a conjugate of glycine and cholate. The extent of hepatocyte damage is reflected in serum CG levels, which can aid in the diagnosis and prognosis of HCC [139]. To monitor CG, Wang et al. grew polyoxometalates (POM, Eu₄W₈) in situ on COFs (EB-TFP). This process maintained the stable crystal structure of the COFs and produced the functional composite material Eu₄W₈@EB-TFP, which enabled rapid (1 min) and trace monitoring with an LOD of 0.024 µg mL^− 1^ [140].

### Other cancers

Colorectal cancer (CRC) is a highly prevalent and lethal cancer among the middle-aged and elderly population. Novel biomarkers are needed for its early clinical diagnosis, and CRC-derived exosomes have demonstrated significant potential. Researchers created a detection method for these exosomes by modifying AuNPs with p-sulfonated cup [4]arene (pSC4) while also loading HRP onto the surface of spherical COFs. This constructed nanoprobe, HRP-pSC4-AuNPs@COFs, has been successfully applied to the electrochemical detection of CRC-derived exosomes. The COFs’ ordered crystal structure and the in situ growth of AuNPs accelerated charge transfer; besides, the high porosity and rigid framework of the COFs allowed for efficient loading and high stability of HRP. The affinity linker pSC4 could specifically recognize amino acid residues on the exosome surface. This method demonstrated good linearity in the 5 × 10^2^ to 10^7^ particles µL^− 1^ range and had an LOD as low as 160 particles µL^− 1^. Testing on clinical serum samples confirmed the method can effectively distinguish between CRC patients and healthy individuals, indicating good clinical application potential (Fig. 7C) [141].

Pheochromocytoma (PCC), a neuroendocrine tumor originating from adrenal medulla chromaffin cells, is represented by catecholamine secretion [143]. The diagnosis of PCC is currently challenging, so CTCs are utilized as effective and representative biomarkers for PCC diagnosis. Developing intelligent, portable, and sensitive devices is crucial for monitoring these CTCs. An integrated electrochemical/visual dual-mode microfluidic device was designed by Liu et al. The device utilized magnetic particles targeted with norepinephrine transporter receptors and somatostatin receptors to capture PCC-CTCs, which overexpressed these receptors. Pt-based COFs (COF@Pt) were then utilized to modify methyl iodobenzylguanidine and octreotide-2,2′,2′′,2′′′-(1,4,7,10-tetrazacyclododecane-1,4,7,10-tetrayl)tetraacetic acid, acting as POD mimics. This approach enhanced the specificity and detection accuracy of CTCs capture and amplified the electrochemical response from H_2_O_2_ reduction. In the presence of PCC-CTCs, TMB oxidized and changed color to allow visual quantification in 5 minutes. A smartphone performed the signal reading, achieving a wide linear range of 2–10^5^ cells mL^− 1^ and an LOD of 1 cell mL^− 1^, making it suitable for early PCC diagnosis (Fig. 7D) [142].

Osteosarcoma (OS) is a primary malignant bone tumor that commonly occurs in children and adolescents. The signaling protein VEGF plays a key role in cancer-associated pathological angiogenesis, so researchers detected VEGF165-overexpressing OS cells (VEGF165^+^ OS-CTCs) as OS markers [144]. A porous nanostructured COFs (M-HO-COF) rich in C = N groups was prepared through the condensation reaction of melamine with cyclohexanone octahydrate. VEGF165-targeted aptamers were efficiently anchored through weak intermolecular forces, which formed aptamer/protein tetramers with high structural stability and specificity. The impedance-based sensor built upon M-HO-COF demonstrated excellent performance in detecting VEGF165^+^ OS-CTCs (LOD as low as 49 cells mL^− 1^). It also achieved an apparent recovery rate of 97.41% in real serum samples [145].

### Pan-cancer biomarkers

**Fig. 8:**
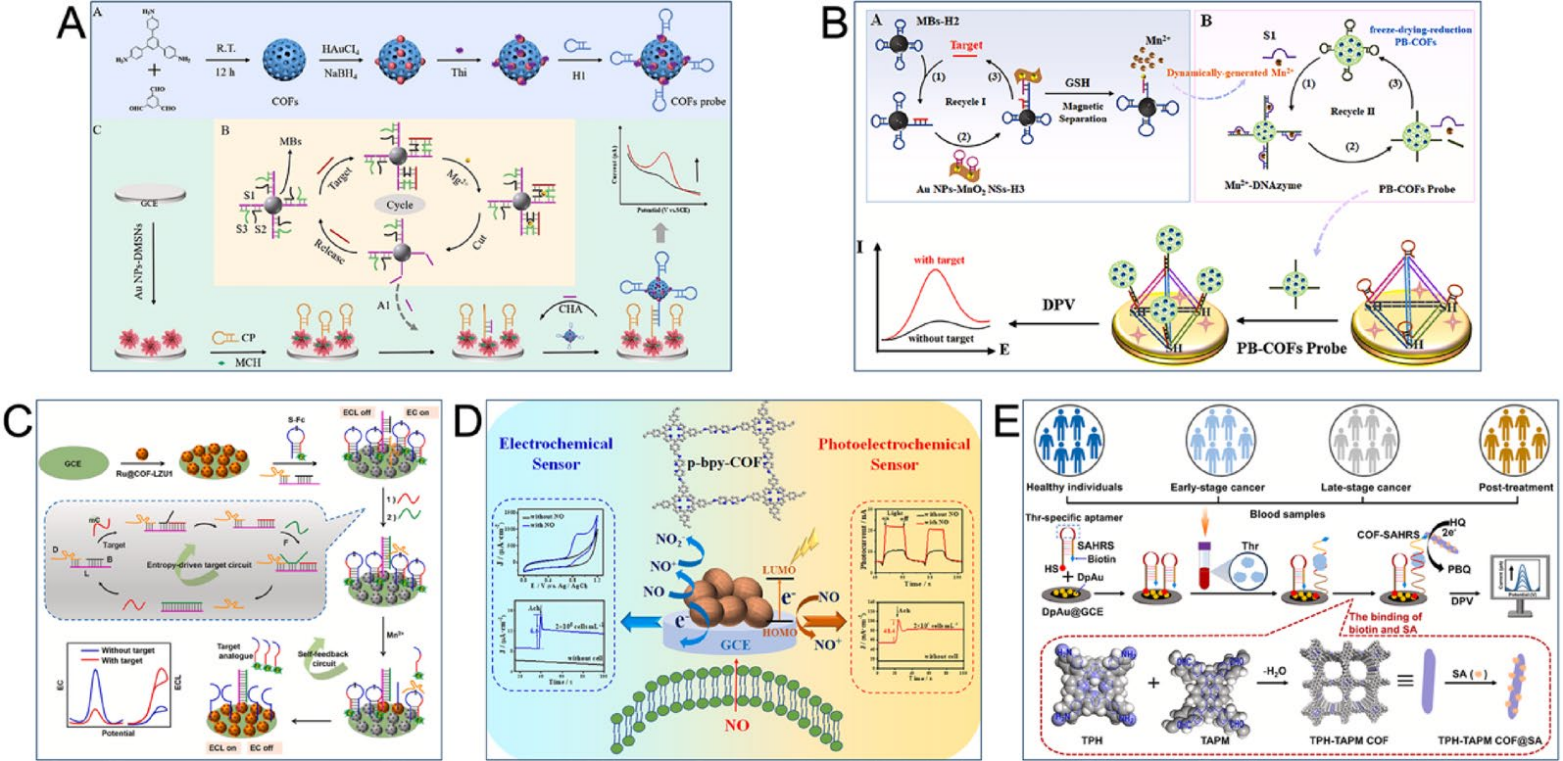
COF-based electrochemical sensors for the detection of pan-cancer biomarkers. (**A**) Illustration of miRNA-155 sensor using magnetic beads-assisted split DNAzyme-driven assembly of functionalized COFs [147]. Copyright 2024, Elsevier. (**B**) Diagram of miRNA-182-5p sensor utilizing target-driven cascade amplified assembly of COFs on m-TDN [148]. Copyright 2024, Elsevier. (**C**) Illustration of the ratiometric ECL/EC sensor for detection of DNA methylation with entropy driven cascaded circuit and Ru@COF-LZU1 accelerator [150]. Copyright 2025, Elsevier. (**D**) Schematic representation of an electroactive and photoactive p-bpy-COF-based bifunctional sensor for in situ analysis of NO from cancer cells [151]. Copyright 2022, Elsevier. (**E**) Diagrammatic sketch of the COF-SAHRS sensor for ultrasensitive detection of Thr [152]. Copyright 2024, Elsevier

Other molecules can also function as pan-cancer biomarkers to assist in cancer diagnosis and treatment. For instance, miRNAs regulate critical biological processes such as gene expression, cell proliferation, and apoptosis; therefore, their abnormal expression is tightly associated with cancer progression. In addition to miRNA-21, other miRNAs such as miRNA-155 and miRNA-182-5p are implicated in the pathological processes of multiple cancers [146]. The CHA strategy has been utilized by researchers to design COF-based sensors for detecting miRNAs. Li et al., for instance, designed a multi-level amplified electrochemical sensor for miRNA-155 detection that used a magnetic bead-assisted, DNAase-driven assembly of functionalized COFs. The synthesized COFs acted as efficient nanocarriers for DNA, while the electroactive molecule thionine (Thi) functioned as signal probes, amplifying the output with excellent hybridization and loading. The operational workflow was simplified by introducing a target-triggered, magnetic bead-assisted DNAase cleavage step. When miRNA-155 was present, it triggered Mg²⁺-dependent DNAase cleavage activity on the magnetic beads. This cleavage generated DNA fragments (A1) that initiated the CHA process, causing a large number of COF probes to assemble on the electrode for further signal enhancement, achieving an LOD of 1.2 fM (Fig. 8A) [147]. The same group also created electroactive COFs for the ultra-sensitive detection of miRNA-182-5p, utilizing a target-driven cascade amplification assembly with TDN with multiple recognition domains (m-TDN). Signal-enhancing probes were prepared by loading electroactive PB and azide hairpin structures H1 onto synthesized COFs through a mild ‘freeze-drying reduction’ method and a CuAAC reaction. Low-abundance miRNA-182-5p was converted into a large amount of Mn²⁺ through target-triggered CHA and GSH reduction, which shifted the detection from a one-to-one conversion to a one-to-many amplification mode. The resulting Mn²⁺-driven DNAase cleavage process induced the assembly of numerous PB-COFs probes onto the m-TDN’s four recognition domains, further amplifying signal intensity. This electrochemical sensor achieved an LOD of 2.5 fM for the ultra-sensitive detection of miRNA-182-5p (Fig. 8B) [148].

DNA methylation is a key epigenetic mechanism where a methyl group binds to the fifth carbon of the cytosine base in CpG dinucleotides. Its abnormal activity is closely linked with tumorigenesis [149]. To monitor DNA methylation levels, Peng et al. developed a dual-mode, ratio-type sensor utilizing ECL and EC. An EC probe (S-Fc) was immobilized on a Ru@COF-LZU1-modified electrode, where S represented the DNA walking substrate. Methylated DNA, after treatment with disulphide, triggered an entropy-driven DNA walking circuit that continuously cleaved S-Fc. The released Fc-labelled fragments contained target analogues which could restart the circuit and activate a self-feedback system. The detachment of Fc from Ru@COF-LZU1 turned off the EC signal and activated the ECL signal. This change produced a significant increase in the ECL/EC ratio, which enabled a sensitive and accurate assessment of DNA methylation levels (LOD of 18 aM) (Fig. 8C) [150].

Nitric oxide (NO), a free radical indicator produced by living cells, functions as a messenger molecule that regulates immune responses and signal transduction [153]. The real-time analysis of NO released from tumor cells is essential for understanding cancer pathogenesis and for early diagnosis. A novel bifunctional sensor based on 2D multifunctional COFs was developed for the sensitive, real-time detection of NO in living cells, combining EC and self-powered photoelectrochemical (PEC) capabilities. Its p-bpy-COF component was synthesized utilizing p-Por-CHO (containing highly conjugated porphyrin ligands) and bpy (featuring electroactive bipyridine groups) as building units. The resulting structure exhibited high conjugation and abundant oxygen vacancies, which improved the electrocatalytic performance for NO and amplified both electrochemical and photonic responses. Moreover, the p-bpy-COF’s low cytotoxicity and good biocompatibility allowed for the in situ detection of NO in live cells. Its large specific surface area and pore size also facilitated rapid diffusion and high adsorption capacity of NO. The sensor demonstrated low detection limits of 17.3 nM and 21.8 nM across the ranges of 0.36–44.3 µM and 5–660 µM, respectively (Fig. 8D) [151].

Cancer-associated thrombosis is a leading cause of cancer-related mortality. During thrombosis, thrombin (Thr) acts in the coagulation cascade and also promotes cancer growth and metastasis [154]. An electrochemical sensor was developed to assess this thrombosis-associated cancer progression, based on a single-stranded aptamer haiDNA response system (SAHRS) integrated with a COFs signal amplification strategy. For signal amplification, COFs were synthesized from TPH (containing porphyrin ligands) and TAPM (with bipyridine groups) and then loaded with streptavidin to form TPH-TAPM COF@SA. In the presence of Thr, the aptamer sequence in the SAHRS specifically bound to it, which induced the denaturation of the haiDNA structure. The interaction between the biotin-labelled SAHRS and TPH-TAPM COF@SA then formed the complete COF-SAHRS sensing platform. This constructed platform detected Thr with a wide linear detection range (10 pM to 20 nM) and an extremely low detection limit (0.17 pM) (Fig. 8E) [152]. The COF-based electrochemical sensors discussed in this review are comprehensively summarized in Table 2.

**Table 2:** An overview of the discussed COF-based electrochemical sensors in this review

Sensor components	Target	Cancer type	Linear Range	LOD	Ref.
MIL-68-NH₂@COF_TP−TA_@CdS	NSE	Lung cancer	0.1 pg mL^−1^–100 ng mL^−1^	0.033 pg mL⁻¹	[86]
COFp-Fepor NH₂-BPA-AuNPs + COFDva-TAB/GCE	NSE	Lung cancer	500 fg mL^− 1^–100 ng mL^− 1^	8.82 J/lg (cNSE/ng mL⁻¹)	[87]
TB-Au-COFs + Au-Ti₃C₂Tx/GCE	CYFRA21-1	Lung cancer	0.5–1.0 × 10⁴ pg mL^−1^	0.1 pg mL^− 1^	[88]
Pep-HRP-COFs (PHCs)	SPARC	Lung cancer	0.5–50 ng mL^−1^	0.23 ng mL^− 1^	[90]
COF@Au-PEI/GCE + haiDNA	EGFR T790M ctDNA	Lung cancer	10^–12^–10^− 7^ M	0.6 pM	[93]
Fe₃O₄@COF/PdAu + CRISPR/Cas12a	EGFR L858R ctDNA	Lung cancer	10^–17^–10^–10^ M	3.3 aM	[94]
NG-PEI-COF_TAPB−TFPB_/GCE + Fe-MOFs	EGFR L858R ctDNA	Lung cancer	100 fM–100 nM	7.65 fM	[95]
P-COF-AuNPs/GCE + His@ZIF-8	EGFR L858R ctDNA	Lung cancer	1 fM–100 nM	0.35 fM	[97]
AuNPs@COF-367/GCE + CRISPR/Cas12a	PD-L1^+^ exosomes	Lung cancer	1.2 × 10^2^–1.2 × 10^7^ particles µL^−1^	38 particles µL^− 1^	[99]
DNA-HRP@COF as probe	PD-L1^+^ exosomes	Lung cancer	2.5 × 10^2^–2.5 × 10^7^ particles µL^−1^	87 particles µL^− 1^	[96]
COFTab-Dva/GCE + electroactive COFTFPB-Thi	CEA	Breast cancer	0.11–80 ng mL^− 1^	0.034 ng mL^−1^	[100]
MIL-156 MOF@COF/GCE	CA15-3	Breast cancer	30–100 nU mL^− 1^	2.6 nU mL^− 1^	[101]
HRP-Ab-AuNPs@COF as probe + APAP as novel medium	HER2	Breast cancer	0.5 pg mL^−1^–100 ng mL^−1^	0.418 pg mL^− 1^	[103]
COFs-AuNPs-Ab-HRP + PEI-MWCNTs/GCE	sPD-L1	Breast cancer	1 pg mL^− 1^–100 ng mL^− 1^	0.143 pg mL^− 1^	[104]
p-COF + carbon dots	VEGF165	Breast cancer	1 pg mL^− 1^–100 ng mL^− 1^	20.9 fg mL^− 1^	[106]
THI/Au/COF as probe + POD-like PW12	CD44	Breast cancer	1 pg mL^−1^–1 µg mL^−1^	0.71 pg mL^− 1^	[108]
COFs/Au/Fc/L1 + TDN/GCE	miRNA-21	Breast cancer	1 fM–10 nM	0.33 fM	[111]
Eu@TAPP-COF + CHA	miRNA-21	Breast cancer	100 aM–100 pM	21 aM	[112]
Pt@COF NSs + ECC	miRNA-21	Breast cancer	100 aM–10 pM	47.5 aM	[113]
COFs-AuNPs/GCE + Cas9n/sgRNA	PIK3CA E545K ctDNA	Breast cancer	10^− 13^–10^− 7^ M	1.76 aM	[116]
Au/Fc@POCs/GCE + MB@COFs as probe	CTCs	Breast cancer	10–10^7^ cells mL^−1^	1 cells mL^− 1^	[114]
Au@COF-LZU1@Ru/GCE + MARS	CTCs	Breast cancer	8–10^5^ cells mL^−1^	2 cells mL^−1^	[117]
HRP@COF as probe	Exosomes	Breast cancer	10^2^–10^8^ particles µL^−1^	80 particles uL^− 1^	[118]
Ru-HAuTiO₂/GCE + Apt-PDA@COF as probe	Exosomes	Breast cancer	3.1 × 10^3^–10^8^ particles µL^− 1^	1.41 × 10^3^ particles uL^− 1^	[119]
EP-TD-COF/GCE + electropositive AuNPs@COFBTT-DGMH	CA125	Ovarian cancer	0.00027–100 U mL^−1^	0.089 mU mL^− 1^	[120]
EP-COF_Dha−Tab_/GCE + electroactive AuNPs@COF_DAAQ−TFP_	CA125	Ovarian cancer	0.01–100 U mL^−1^	0.0067 U mL^−1^	[121]
Ce-MOF/TPN-COF/CNT/GCE	CA125	Ovarian cancer	0.0001–100 U mL^−1^	0.000088 U mL^− 1^	[122]
TMTA-PBA-COF as probe	SA	Ovarian cancer	1–500 µM	0.45 µM	[124]
TpPa-1 COFs@Dye as probe	SA	Ovarian cancer	10^− 8^–10^− 2^ M	7.08 × 10^− 9^ M	[125]
TCPB-DMTA-COF as probe	ALP	Ovarian cancer	0.01–100 U L^−1^	6 × 10^− 3^ U L^− 1^	[127]
2D COFs/ITO + AgInS₂ QDs	CEA	Cervical cancer	50 fg mL^−1^–0.5 µg mL^−1^	3.25 fg mL^− 1^	[130]
2D crystal COF4/SPCE	GSH	Cervical cancer	1–1000 µM	0.191 µM	[128]
MB@DNA-COF as probe	Exosomes	Cervical cancer	10^4^–10^7^ particles µL^− 1^	9661 particles µL^− 1^	[129]
DNA-COF-HRP-cholesterol-labelled DNA (HDCs)	Exosomes	Cervical cancer	10^4^–10^7^ particles µL^− 1^	7668 particles µL^− 1^	[132]
Au@PDA@BCN/GCE + AuPt@MnO₂@COF	PSA	Prostate cancer	0.00005–10 ng mL^−1^	16.7 fg mL^−1^	[133]
POD-like Fe₃O₄@COF@Os	PSA	Prostate cancer	0–250 ng mL^−1^	3.83 pg mL^−1^	[134]
hexagonal 2D imine-based COFs	SAR	Prostate cancer	0.5–700 fM/ 10 pM–0.12 nM	0.15 fM	[136]
Apt/COF@CNF/AuNPs/GCE + MIP	SAR	Prostate cancer	500 fM–50 pM/ 50–350 pM	0.166 pM	[137]
Fe₃O₄NPs@COF/AuNPs/GCE + MNPs@SiO₂@TiO₂	AFP	HCC	0.01–1 pg mL^− 1^	3.30 fg mL^− 1^	[138]
Eu₄W₈@EB-TFP COFs	CG	HCC	10^− 8^–10^− 3^ M	0.024 µg mL^− 1^	[140]
HRP-pSC4-AuNPs@COFs as probe	CRC derived-exosomes	CRC	5 × 10^2^–10^7^ particles µL^−1^	160 particles µL^− 1^	[141]
POD-like COF@Pt-MIBG-DOTA + magnetic particles	PCC-CTCs	PCC	2–10^5^ cell mL^−1^	1 cells mL^− 1^	[142]
M-HO-COF/AuE	VEGF165^+^OS-CTCs	OS	10^2^–10^5^ cells mL^−1^	49 cells mL^− 1^	[145]
H1/Thi/AuNPs-COFs + CHA	miRNA-155	Pan-cancer	10 fM–5 nM	1.2 fM	[147]
PB-COFs + CHA	miRNA-182-5p	Pan-cancer	10 fM–100 nM	2.5 fM	[148]
S-Fc/Ru@COF-LZU1/GCE	DNA methylation	Pan-cancer	50 aM–50 pM	18 aM	[150]
electroactive p-bpy-COF/GCE	NO	Pan-cancer	0.36–44.3 µM/ 5–660 µM	17.3 nM/ 21.8 nM	[151]
TPH-TAPM COF@SA + SAHRS	Thr	Pan-cancer	10 pM–20 nM	0.17 pM	[152]

## Conclusion and perspectives

This review comprehensively summarizes the latest research progress over the recent five years in cancer-related COF-based electrochemical sensors. Tumor markers are crucial in early cancer diagnosis, treatment effectiveness assessment, recurrence monitoring, and prognosis determination. Their low abundance in body fluids has focused research on high-performance detection, for which COF-based electrochemical sensors are utilized to target proteins, nucleic acids, CTCs, and EVs. Diverse synthetic methods produce COFs with unique physicochemical properties and allow them to enhance electrochemical sensors post-functionalization. The integration of DNA strategies, enzyme or enzyme-like catalytic amplification methods, electrocatalytic reactions, microfluidic technology, and other reaction strategies enables COF-based electrochemical sensors to demonstrate excellent specificity and accuracy in analyzing various tumor markers. This paper outlines the synthesis and functionalization strategies for COFs, discusses common electrochemical reaction mechanisms, and offers a detailed introduction to the application of COF-based electrochemical sensors in liquid biopsy for cancer. While COF-based electrochemical sensors have made significant progress in the quantitative detection of tumor markers over the past five years, they still face numerous challenges that require attention.

### Advantages and challenges of COFs in electrochemical sensor applications

The unique properties of diverse COFs enable their varied applications in the aforementioned tumor marker detection. By incorporating redox units, electroactive COFs enhance electronic transport capabilities and significantly improve the sensitivity of electrochemical sensors [87]. Functionalized COFs leverage their modifiable pore structures for immobilizing recognition units, DNA, or enzymes, which enables specific target capture and improved biocompatibility [86, 129]. Nanocomposite COFs construct multifunctional detection platforms through synergy with materials such as metal nanoparticles [140]. These COF materials exhibit immense potential for the enhancement of electrochemical sensor performance.

Two key factors contribute to the exceptional analytical capabilities of COF-based electrochemical sensors. The first factor is the advantages of electrochemical methods, which include rapid response, ease of operation, low cost, and high sensitivity. The second involves the unique properties of COFs: (1) Schiff base condensation reactions are the primary method for synthesizing the COFs utilized in these sensors, a method represented by a simple preparation process and products with high crystallinity and robust stability. Functionalization of the synthesized COFs’ basic structure is achieved through surface modification, in situ synthesis hybridization, and self-assembly. The resulting synergistic effects significantly enhance COFs’ performance and offer insights into their stable, large-scale production. (2) In electrochemical analysis, COFs are generally employed as recognition units, signal converters, loading platforms, enrichment agents, or adsorbents. The porous structure and heteroatoms of COFs can capture targets effectively through non-covalent interactions for accurate recognition. Excellent mass transfer efficiency and conductivity result from their ultra-microporous structure, π-π conjugated framework, and ordered channels, while their large specific surface area offers extensive electroactive regions and high loading capacity. After hybridization, COFs gain novel properties such as electrocatalysis and enzyme catalysis, creating a foundation for the accurate control of micro-reactions. Besides, the excellent biocompatibility and high stability of COFs allow them to protect modified active molecules (such as antibodies and enzymes) through hybridization with specific molecules, which offers strong support for developing stable, recyclable electrochemical sensors. (3) Various carefully designed reaction strategies are employed to improve sensor performance. These strategies include DNA-based approaches such as CRISPR/Cas12a and CRISPR/Cas9n systems, CHA strategies, DNA walkers, MARS, TDN, etc. Other methods such as mild and efficient CuAAC reactions, enzyme/enzyme-like enhancement methods, electrocatalytic or ECC redox methods, magnetic enrichment methods, and MOF/COF synergistic strategies also lay the foundation for enhanced sensor function. Therefore, electrochemical sensors based on COFs demonstrate excellent analytical performance for biomarkers ranging from nucleic acids and proteins to cells and vesicles. This capability is highly advantageous for their clinical application and widespread adoption.

Several challenges remain for COF-based electrochemical sensors, notwithstanding their outstanding performance and immense potential. First, chemical stability represents a critical bottleneck as numerous imine-based COFs are subjected to hydrolysis in aqueous solutions and the slightly acidic TME, which leads to structural collapse and performance degradation. Second, prominent reproducibility issues occur since the crystallization process is highly sensitive to conditions, resulting in significant performance differences between batches. In addition, inefficient assembly and poor reproducibility often result from the relatively low affinity of COFs, creating a substantial gap between laboratory-scale and industrial-scale production. Most critically, real-sample applicability is a major concern, as interfering substances in complex clinical samples may clog COFs pores, while systematic evaluation of the biocompatibility and potential cytotoxicity of COFs has yet to be performed. Technical challenges also require thorough consideration as COF-based electrochemical sensors transition from the laboratory to commercialization. Critical issues are improving the conductivity of COFs prepared from non-conductive components, optimizing modification strategies, and signal transmission in electrochemical sensors.

Advancing the clinical application of COF-based electrochemical sensors requires future work to focus on several key aspects. (1) COFs synthesis methods should be studied and optimized to achieve simple conditions, high crystallinity and robust stability, which can explain the relationship between structure and performance. (2) Functionalization strategies also need improvement to optimize COF affinity, active sites, specific surface area, interference resistance, and conductivity, while enhancing biocompatibility simultaneously. (3) The development of COF-based nano-hybrid materials could enhance synergistic potential, thereby improving the selectivity and accuracy of electrochemical sensors and offering a superior, tunable platform. (4) Since various tumor markers have different clinical significance, it is necessary to develop sensors capable of detecting multiple tumor markers to achieve simultaneous multi-target detection, enabling collaborative patient assessment and meeting clinical multi-target analysis requirements. (5) For applicability in complex clinical samples, diverse electrochemical reaction strategies must be designed to enhance the sensor’s specific recognition capability, and microfluidic devices can be utilized to reduce matrix interference for more accurate clinical detection.

### Clinical translation prospects of COF-based electrochemical sensors for cancer diagnosis and surveillance

#### Clinical translation pathway for COF-based electrochemical sensors

For electrochemical sensors, commercialization and widespread clinical application depend on a systematic process for translating scientific discoveries into reliable medical products [155]. This pathway starts with proof-of-concept validation under ideal laboratory conditions and then continues with engineering and preclinical validation in real clinical settings. Ensuring product performance consistency requires the process to progress through the following multicenter, large-sample prospective clinical studies, production scaling, and regulatory approval. For cancer diagnosis and monitoring, COF-based electrochemical sensors also follow a progressive value-added process. This process begins with the sensors representing a powerful complement to existing “gold standard” pathological biopsies to enhance diagnostic and triage efficiency, then functioning as dynamic “monitoring instruments” for managing treatment effectiveness and recurrence, and finally enabling personalized accuracy interventions. Priorities for laboratory research and engineering must include sensor stability, sensitivity, reproducibility, and digitalization, as well as low-cost materials and simplified manufacturing methods [156]. Electrochemical sensors can be integrated with microfluidics, wearables, or portable devices to enable rapid data readout and analysis. Such integration facilitates POCT, particularly in resource-constrained settings, and holds promise for transforming cancer diagnosis and health management paradigms [157, 158]. The use of technologies such as SPCEs and microfluidics enables batch integration and production [128]. For their application in POCT, however, several challenges must be addressed: signal conversion, amplification, and readout; electronic component packaging; single-board integration of reaction modules; development of simple, stable microfluidic systems; and device intelligence. Electrochemical sensors integrated into wearable platforms are more susceptible to variations in pH, temperature, and ionic strength, while mass-produced integrated sensors also face challenges with storage and long-term stability. To enhance sensor stability and repeatability, we propose utilizing electrocatalysis or nanozymes as alternatives to enzymes and constructing multiplexed detection arrays.

Certain electrochemical sensors have already gained regulatory approval or commercialization. For instance, ECL immunoassay analyzers (e.g., Abbott ARCHITECT i2000SR, Snibe MAGLUMI X8) are widely utilized in hospitals to detect tumor markers such as CEA, PSA, and AFP. Portable blood gas and electrolyte analyzers (e.g., Seamaty SG1) also target the POCT market by utilizing dry electrochemical methods and microfluidics to monitor blood electrolytes and gases. Most research, however, remains focused on sensor design, such as utilizing electropositive AuNPs@COFBTT-DGMH to detect the ovarian cancer marker CA125 or developing sensors based on COF@CNF/AuNPs and MIP for the prostate cancer marker SAR [120, 137]. These studies are at the prototype stage and therefore lack reliable data from large-scale clinical trials, so the variability and reproducibility of such sensors in clinical settings have yet to be evaluated. While electrochemical sensors frequently exhibit excellent performance in controlled laboratory environments, significant performance gaps can appear during actual clinical validation. This gap primarily results from the conflict between ideal conditions and complex clinical settings. Such complexities include matrix effects in clinical samples (e.g., lipids, proteins, drug interferents), potential interferents derived from patients (e.g., heterophilic antibodies, rheumatoid factors), and uncontrollable variables such as user operation and environmental fluctuations, none of which can be represented in standard laboratories. During clinical validation, these factors can collectively cause signal drift, cross-reactivity, and reduced reliability. Narrowing this gap to clinically acceptable levels requires forward-thinking scientific design, comprehensive validation, and iterative optimization, which can ensure that sensors deliver safe, effective, and reliable diagnostic information for cancer patients.

The core of clinical translation for COF-based electrochemical sensors is the establishment of standardized methodologies. This process involves evaluating safety and effectiveness in preclinical studies and clinical trials and establishing quality systems with regulatory frameworks to ensure standardized clinical use in later stages. For sensors intended for POCT, regulatory considerations exhibit various aspects; they consist of analytical performance, personnel qualifications, quality control, data traceability systems, and clear instructions for use. Adherence to these points ensures the credibility of individual test results and compliance throughout the clinical workflow, which finally allows for safe and effective support in clinical decision-making [159]. Among these considerations, establishing reference intervals and calibrating for different sample types are critical factors that affect quality control. Establishing reference intervals requires selecting appropriate sample types, followed by data analysis and validation after sensor detection, which confirms the method’s applicability in specific populations. Inherent variations exist among serum, plasma, whole blood, urine, saliva, and other sample types. Standardization therefore necessitates that sensors be equipped with additional electronic components for the dynamic calibration of pH levels, red blood cell concentration, flow rate, and other parameters across different samples.

#### Challenges in clinical translation of COF-based electrochemical sensors

In the field of liquid biopsy, COF-based electrochemical sensors demonstrate immense research potential and broad development opportunities. Their clinical translation, however, faces multidimensional challenges. At the regulatory approval level, demonstrating sufficient evidence constitutes the core challenge. Proving analytical and clinical effectiveness in high-risk medical decision-making requires rigorous data that is highly consistent with current clinical “gold standard” pathological biopsies—a time-consuming and costly process. Practical application validation faces a gap between ideal laboratory and variable clinical settings. In clinical settings, complex sample matrices and fluctuating operational conditions test sensor robustness and result reliability. Component stability is central for commercialization. The activity decay of bio-recognition elements directly limits product sensitivity and shelf life; extending shelf life is also closely related to production scaling. A major challenge remains in ensuring consistent bio-modification across every sensor during high-yield, low-cost mass manufacturing. In addition, POCT viability relies on simplifying complex procedures significantly and incorporating intelligent error detection to reduce risks from non-professional users [160]. Finally, all these efforts must be translated into patient acceptance, which comprises tolerance for testing discomfort, accurate interpretation of results, and willingness to pay. This forms the final loop in the commercialization chain.

COF-based electrochemical sensors also face practical deployment barriers. Technologically, the long-term stability of bioelements and batch-to-batch consistency constrain large-scale adoption. Clinically, integrating sensor data with existing diagnostic workflows is a struggle, as it lacks matching clinical pathways and accountability standards. The regulatory frameworks for evaluating innovative combination products and algorithms are still underdeveloped. In addition, lagging payment mechanisms directly constrain adoption, as traditional fee-for-service models struggle to cover the upfront monitoring and health management value. User acceptance presents an end-user barrier; real-world effectiveness is jointly impacted by the data interpretation capabilities of healthcare providers and the technical acceptance of patients. To overcome these interconnected obstacles, cross-disciplinary systemic solutions are required.

Besides, cost remains the most critical issue for the clinical translation of COF-based electrochemical sensors. To achieve economies of scale, considerations include reducing core component manufacturing costs through technological innovation and optimizing production processes. Establishing a tiered product system would offer full-featured solutions for premium markets while developing affordable versions for primary care settings. A shift from per-service fees to long-term health outcome-based compensation requires actively exploring value-based payment models. Finally, key technologies can be integrated into public health initiatives through public-private partnerships, which facilitates the transition from high-end technology to accessible tools.

#### Interdisciplinary collaboration accelerates clinical translation of COF-based electrochemical sensors

ML and AI are emerging as transformative forces in cancer diagnostics. These technologies construct high-precision predictive models for early tumor screening, subtype diagnosis, and prognosis prediction by deeply mining multi-omics data, medical imaging, and electronic health records. The integration of ML/AI with COF-based electrochemical sensors creates new opportunities for intelligent diagnostics [161]. ML allows for the design of COF materials tailored to the TME and optimizes signal processing, which enhances detection accuracy and reliability while achieving accurate multi-target quantification in complex samples. With real-time detection results, AI dynamically adjusts sensor parameters to enable self-optimizing and self-calibrating intelligent detection processes. Beyond enhancing sensor performance, ML/AI offers traditional sensors with unprecedented capabilities for data analysis and decision-making, offering new technical pathways for next-generation intelligent diagnostic systems.

In summary, the clinical application of COF-based electrochemical sensors remains challenging; however, the rational design of COF materials and continuous sensor optimization hold promise for overcoming these clinical application bottlenecks. Future research will focus on achieving ultra-sensitive detection utilizing nano-interface engineering and signal amplification strategies, overcoming wearable technology limitations for capturing dynamic monitoring data, and enabling intelligent multi-marker analysis through the integration of microfluidics with ML/AI. Innovative manufacturing processes will finally drive technological accessibility, which will elevate the value proposition from an auxiliary diagnostic tool to a comprehensive accuracy tumor management platform. Such progress will revolutionize early cancer diagnosis and longitudinal surveillance paradigms and contribute to an improved quality of life for cancer patients.

## Data Availability

No datasets were generated or analysed during the current study.
